# Intra-articular injection of allogeneic canine adipose-derived stem cell enriched secretome in dogs undergoing tibial plateau leveling osteotomy: a prospective double-blind and randomized pilot trial

**DOI:** 10.1007/s11259-026-11343-9

**Published:** 2026-06-13

**Authors:** Alefe Luiz Caliani Carrera, Bruno Watanabe Minto, Brenda Mendonça Alcântara, Daniel Alexandre Coimbra Previtali, Rodrigo Carvalho Souza Faustino, Angélica Barreto Leite Tavares, Danuta Pulz Doiche, Camila Nogueira, Hilana Santos Sena Brunel, Rinaldo Wellerson Pereira, Marcelo Meller Alievi, Luis Gustavo Gosuen Gonçalves Dias, Patricia Furtado Malard

**Affiliations:** 1 Department of Veterinary Clinics and Surgery, School of Agricultural and Veterinary Studies (FCAV), São Paulo State University (UNESP), Access Road Prof. Paulo Donato Castellane s/n, Jaboticabal, State of São Paulo, Jaboticabal, SP 14884-900 Brazil; 2 Postgraduate Program in Genomic Sciences and Biotechnology, Catholic University of Brasilia (UCB), Brasília, DF Brazil; 3 Department of Animal Medicine, Faculty of Veterinary Sciences (FAVET), Federal University of Rio Grande do Sul (UFRGS), Porto Alegre, (RS) Brazil

**Keywords:** Accelerometry, Adjuvant therapy, Cranial cruciate ligament, Osteoarthritis, Veterinary surgery

## Abstract

This pilot study evaluated the short- and mid-term outcomes of intra-articular allogeneic canine adipose-derived stem cell (ADSC) enriched secretome in dogs undergoing tibial plateau levelling osteotomy (TPLO), focusing on postsurgical recovery and osteoarthritis (OA) progression. Twenty client-owned dogs were randomly assigned (*n* = 10 per group) to a control group (CG), receiving placebo, or a treated group (ADSC-Se), receiving 250 µg of ADSC-derived enriched secretome intra-articularly at the end of TPLO. Lameness, stifle pain, accelerometry, and OA-related changes assessed by radiography and articular ultrasonography scores were evaluated for up to 150 days postoperatively. Median lameness (3 to 1) and stifle pain (2 to 0) scores decreased in ADSC-Se group at 15 days postoperatively, whereas similar improvements in CG occurred later (60 and 90 days postoperatively, respectively). No differences occurred in accelerometry outcomes. Radiographic assessment showed progressive median OA in the CG (1 to 2), whereas OA scores decreased in ADSC-Se group (1 to 0) from 15 days postoperatively and remained stable thereafter. Ultrasonography revealed reduced median joint effusion scores in ADSC-Se group (1 to 0) from 30 days postoperatively, with improvement in cartilage appearance from thin to normal, whereas CG showed no effusion improvement and cartilage deterioration. Radiographic bone healing scores at 30 days post-TPLO were higher in the ADSC-Se group, with mean differences of 1.9 (10-point scale) and 1 (5-point scale). Intra-articular ADSC-enriched secretome was associated with faster postoperative clinical improvement and reduced OA progression, suggesting its potential as a biologic adjunct to TPLO.

**Supplementary Information:**

The online version contains supplementary material available at https://doi.org/10.1007/s11259-026-11343-9.

## Introduction

Cranial cruciate ligament disease (CCLD) can progress to secondary osteoarthritis (OA) and is one of the most common causes of pelvic limb lameness in dogs (Malek et al. 2020; Spinella et al. 2021; Villatoro et al. 2023). Despite decades of research, no universally accepted treatment prevents degenerative joint disease (DJD) in CCLD patients (Ballagas et al. 2004; Glyn-Jones et al. 2015; Kuyinu et al. 2016). Furthermore, no gold-standard treatment for CCLD exists due to limitations in current techniques. Surgical interventions, particularly proximal tibial osteotomies, have shown favorable results in promoting dynamic stifle stabilization, reducing joint instability, and yielding satisfactory clinical outcomes (Brioschi and Arthurs 2021).

Tibial plateau leveling osteotomy (TPLO) is the most widely used technique for treating CCLD worldwide, aiming to achieve dynamic stifle stabilization by leveling the tibial plateau (Slocum and Slocum 1993). However, even after 30 years of implementation and widespread adoption as one of the most extensively investigated surgical techniques, TPLO has not proven to be flawless. Some patients experience suboptimal outcomes, particularly regarding short- and mid-term clinical recovery, which may extend beyond 30 weeks before achieving full restoration of limb function (Heidorn et al. 2018). Furthermore, OA progression is commonly observed postoperatively (Shimada et al. 2020), frequently attributed to residual stifle instability (Tinga et al. 2020). Paradoxically, early weight bearing on the affected limb is encouraged to mitigate muscle atrophy and accelerate clinical recovery (Monk et al. 2006). In this context, adjuvant therapies are warranted to reduce inflammation and enhance surgical outcomes.

The use of platelet-rich plasma and hyaluronic acid has not effectively enhanced clinical recovery or control of OA progression when compared with TPLO alone (Volz et al. 2024). Similarly, the oral administration of nutraceuticals has been proven ineffective in reducing the postsurgical progression of radiographic OA (Martini et al. 2017), highlighting the absence of effective surgical adjuvants. In this context, the use of extracellular vesicles and protein enriched secretory products from canine adipose-derived stem cells (ADSC), collectively known as the enriched secretome, may be considered promising adjuvant therapy due to their anti-inflammatory, antioxidant, and cartilage-regenerative properties (Gowen et al. 2020; Tieu et al. 2020). Enriched secretome may offer distinct advantages over whole ADSCs by concentrating bioactive molecules, thereby enhancing protein modulation and interleukins signaling within the articular environment (Bousnaki et al. 2020; Li et al. 2022). Despite the favorable outcomes reported in previous studies on the use of ADSC-secretome (ADSC-Se) in veterinary patients (Bousnaki et al. 2020; Sharifian et al. 2017; Tieu et al. 2020), a paucity of evidence supporting its use in canine clinical models of OA is observed.

Therefore, intra-articular injection of ADSC-Se is hypothesized to effectively promote early clinical recovery and reduce mid-term OA progression following TPLO in dogs due to its anti-inflammatory and antioxidant properties. Given the need for better outcomes in TPLO-treated patients and the potential benefits of ADSC-Se, assessing clinical and imaging parameters is crucial to validating its intra-articular efficacy in enhancing mild- to mid-term recovery and controlling OA in dogs undergoing TPLO. Therefore, this study was designed to address these objectives.

## Methods

The international animal welfare guidelines were adhered to in all procedures, and the Animal Ethics Committee of FCAV UNESP approved this study under protocol number 9138/22. All dog owners accepted and provided informed consent for participation in the study. As the animals enrolled in this study were client-owned pets, they were not euthanized following the procedures, and all postoperative care was conducted under standard clinical guidelines to ensure their well-being.

### Inclusion criteria and study design

This double-blind (for owners and assessors), placebo-controlled and prospective trial was conducted on client-owned dogs diagnosed with CCLD and undergoing TPLO at a referral teaching veterinary hospital. A total of 20 patients were enrolled. Owing to the absence of previous clinical data, this study was conducted as a pilot trial, with sample size (*n* = 20) defined based on feasibility and case availability. These patients were diagnosed with unilateral CCLD, confirmed via cranial drawer and tibial thrust tests, and underwent TPLO. No breed restrictions were applied. The inclusion criteria consisted of age between 1 and 7 years, body weight between 10 and 45 kg, absence of clinical or laboratory evidence of systemic diseases, no DJD in other pelvic limb joints, and a tibial plateau angle (TPA) between 15° and 35°. The exclusion criteria were chronic endocrine disorders, previous bone fractures in the pelvic limbs, DJD in the hip or tarsal joints, severe soft tissue injuries in the pelvic limb, or limb amputation.

The patients were randomly assigned to two experimental groups (control and treatment), each consisting of 10 animals, in a parallel design. Randomization was conducted by drawing lots, with the drawn number corresponding to the vial containing either the treatment or placebo, which was revealed only at the end of the experiment.

### Surgical procedure and postoperative care

Mediolateral radiographs of the tibia were reviewed to measure the preoperative TPA. Surgical planning for TPLO was performed using the vPOP Pro software (VETSOS Education Limited) to achieve a resulting TPA of 5°. For surgical procedure, food was withheld for 12 h prior to surgery, while water was allowed until premedication. Methadone (0.3 mg/kg) was administered intramuscularly 20–30 min before anesthesia induction. Anesthesia was induced via intravenous administration of propofol (2 mg/kg) in combination with ketamine (1 mg/kg) and lidocaine (1.5 mg/kg) and maintained with isoflurane in oxygen. Lactated Ringer’s solution (5 mL/kg/h) was administered throughout general anesthesia. The lumbosacral joint region was aseptically prepared, and bupivacaine (0.5 mg/kg) along with lidocaine (4 mg/kg) was administered epidurally. Cephalothin (30 mg/kg) was given intravenously at induction and repeated 1.5 h later. The TPLO procedure was performed according to Slocum and Slocum (1993) by the same surgical team. Arthrotomy was performed after TPLO via a medial parapatellar incision to assess the meniscal integrity and to grade macroscopic DJD as mild, moderate, or severe. The tear in cases of meniscal tear was classified, and partial meniscectomy was performed, following standard protocol (Thieman et al. 2010). Surgical implants (plates and screws) were standard models. Postoperatively, the patients received an analgesic protocol consisting of methadone (0.2 mg/kg intramuscular, single dose) and tramadol hydrochloride (4 mg/kg every 8 h), as well as carprofen (2.2 mg/kg every 12 h) for 7 days. In addition, antibiotic therapy with amoxicillin and potassium clavulanate (22 mg/kg every 12 h) was administered for 10 days. Postoperative assessments were conducted in the short- and medium-term periods, and complications were classified as minor, major, or catastrophic (Cook et al. 2010).

### Intra-articular treatment

The treated group (ADSC-Se) was administered an intra-articular injection of sterile allogeneic canine ADSC-Se, whereas the control group (CG) received an intra-articular injection of a placebo. Intra-articular injections were administered intraoperatively after completing the arthrotomy. The ADSC-Se group was treated with liquid samples of the ADSC-enriched secretome, standardized to a protein concentration of 250 µg, and diluted in Ringer’s lactate to a final volume of 0.7 mL. The CG was administered 0.7 mL of sterile Ringer’s lactate.

### ADSC-enriched secretome acquisition

The canine ADSCs and the secretome production process used in this study were provided by BioCell by Vetnil Company. The ADSCs were obtained from the adipose tissues of three healthy female canine donors. The tissues samples were washed four times with Dulbecco’s modified phosphate-buffered saline and then fragmented, followed by enzymatic digestion in a solution of collagenase and hyaluronidase for 30 min at 38.5 °C. Subsequently, the mononuclear cells obtained from each donor were placed separately with 3 × 10⁵ cells per T75 culture flask with Dulbecco’s modified eagle medium (DMEM) with phenol red and 10% of fetal bovine serum (FBS) at 37.5 °C, in a controlled atmosphere with 5% CO₂ for 7 days. Afterward, the cells were transferred to larger flasks and cultured until they reached 90% confluence.

Subsequently, the cells were cultured in cell culture flasks with DMEM medium supplemented with 10% FBS. Upon reaching 70% confluence, the culture medium was replaced with DMEM without phenol red and FBS. The cells remained in culture for 2 to 5 days until they reached 90% confluence, at which point the culture medium was harvested for secretome production and average cellular counting was assessed for each batch. In addition, the ADSCs cultured were trypsinized, aliquoted and cryopreserved in liquid nitrogen (-196 °C). After that, ADSC undergone characterization and quality control as detailed in the supplementary material (Online Resource 1). 

The mean cell counts at the completion of ADSC culture and supernatant collection were 0.95 × 10^6^ ± 0.17 × 10^6^ cells/mL, 1.03 × 10^6^ ± 0.17 × 10^6^ cells/mL, and 0.96 × 10^6^ ± 0.18 × 10^6^ cells/mL for each batch, respectively. The culture medium was harvested from the cellular culture and transferred to 50 mL conical tubes for centrifugation at 2000 g for 15 min to remove cellular debris. Then, apoptotic bodies were removed in the second centrifugation performed at 17,000 g for 40 min. Larger particles were removed from the supernatant collected and filtered using a 0.22 μm syringe filter. Then, 15 mL of the resulting solution was collected and placed into a 100 kDa ultrafiltration device, where it was re-centrifuged at 4000 g for 15 min, yielding an enriched secretome medium. This content was aspirated with a micropipette (p200) and transferred to a 1.5-mL microtube, totaling approximately 250 µL of the enriched compound. These procedures were performed for each batch from the donors, and the three enriched compounds from each donor were combined to create a pooled enriched secretome. The pool was then subjected to protein quantification using the Bradford method (Kruger 2009), with three samples of 10 µL collected and placed into 200 µL tubes. The results were recorded in an Excel spreadsheet to calculate the standard curve and the determination of protein concentration in µg/mL of the enriched sample, enabling the preparation of the final sample containing 250 µg in 0.7 mL of Ringer’s lactate solution. Each sample was prepared, aliquoted into pellets, and cryopreserved in liquid nitrogen (− 196 °C) until use. For application, the samples were thawed at 38 °C for 20 s.

### Outcomes

The patients were assessed pre- and post-operatively through accelerometry, clinical gait analysis, stifle pain evaluation, radiographic assessment of stifle OA progression, and articular ultrasonography. Preoperative assessment was performed between 10 and 20 days preoperatively. Postoperative evaluations were performed at the following time points: (1) 24 h, (2) 48 h, (3) 15 ± 5 days, (4) 30 ± 5 days, (5) 60 ± 5 days, (6) 90 ± 5 days, and (7) 120–150 days. Assessments within the first 48 h postoperatively were performed via video recordings of the patient at home, each lasting at least 20 s while walking, except for accelerometry, which was attached to patients during every assessment. Other evaluations were conducted through in-person follow-ups.

#### Accelerometry assessment

Accelerometry was performed with an ActiGraph wGT3X-BT accelerometer (ActiGraph LLC, Florida, USA) positioned on the ventral region of the dogs’ necks and was conducted for activity level analysis at all evaluation time points. Data were collected over 24 h at 15-s intervals, with the device recording vector counts in three axes. After completing each recording period, the data were retrieved using ActiLife 6 software (ActiGraph LLC, Florida, USA), which processed the tri-axial vector counts to generate a resultant vector. Based on the counts per minute (cpm) of the resultant vector over 24 h, activity levels were categorized as sedentary (≤ 1351 cpm), moderate (1352–5695 cpm), or vigorous (≥ 5696 cpm; Schuster et al. 2023), and the time spent in each category was summarized.

#### Lameness assessment

A single evaluator evaluated the subjective limb function at all pre- and post-operative time points using a five-point lameness scale (Cook et al. 1999). Scores ranged from 0 to 4 based on the degree of support provided by the affected limb: 0, the absence of visual clinical lameness; 1, mild lameness during weight bearing; 2, moderate lameness during weight bearing; 3, severe lameness during weight bearing; and 4, complete functional loss without support.

#### Stifle pain assessment

Orthopedic examination was conducted (Jeong et al. 2021) during the preoperative period and at postoperative time points (3) through (7). Stifle pain was graded on a five-point scale during limb extension and flexion and during digital palpation of the medial and lateral stifle surfaces: 0, absence of painful sensitivity; 1, mild painful sensitivity with limb traction in response to the stimulus; 2, moderate painful sensitivity with limb traction accompanied by vocalization; 3, severe painful sensitivity with limb traction, vocalization, and attempted biting; and 4, severe pain with refusal to allow joint contact.

#### Radiographic assessment

Radiographic assessment was performed preoperatively and at postoperative time points (3) through (7) following TPLO using mediolateral and craniocaudal projections of the stifle that included the entire tibia. A single examiner with expertise in diagnostic imaging conducted all evaluations. A six-point scale for stifle OA was adopted (Moore et al. 2020): 0, absence of joint effusion or osteophytosis; 1, initial acute OA with joint effusion but no osteophytes; 2, mild joint disease with osteophytosis of the patella and the edges of the patellar groove of the femoral trochlea; 3, moderate joint disease with small osteophytes in the patella, patellar groove edges, femoral condyles, fabella, articular margins of the tibial plateau, and fibular head; 4, moderate-to-severe joint disease with medium to large osteophytes in the patella, patellar groove edges, femoral condyles, fabella, articular margins of the tibial plateau, and fibular head, in addition to moderate subchondral sclerosis; and 5, severe joint disease with large osteophytes in the patella, patellar groove margins, femoral condyles, fabella, articular margins of the tibial plateau, and fibular head (including the intercondylar notch), accompanied by moderate calcification and subchondral sclerosis. Furthermore, the time required for complete ossification was recorded, and bone consolidation was graded at 30 days postoperatively using the previously established 10-point and 5-point scales (Kieves et al. 2015).

#### Ultrasonographic assessment

**Fig. 1:**
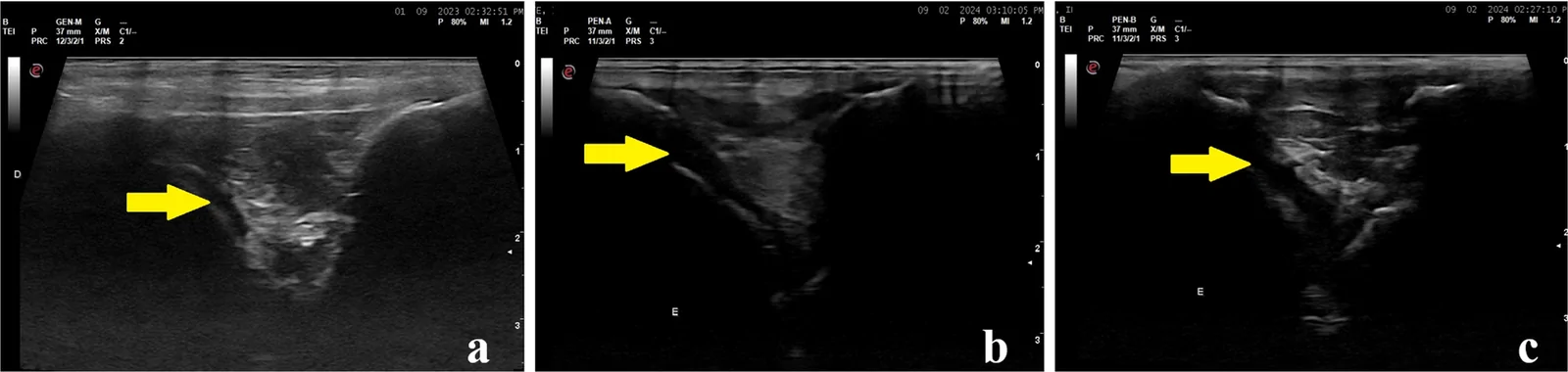
Ultrasonography assessment of joint effusion (arrow) in the stifle joint of dogs. Longitudinal view via the craniomedial approach, comprising the medial femoral condyle, infrapatellar region, and tibial articular surface. Examples show mild effusion (**a**), moderate effusion (**b**) and severe effusion (**c**)

**Fig. 2:**
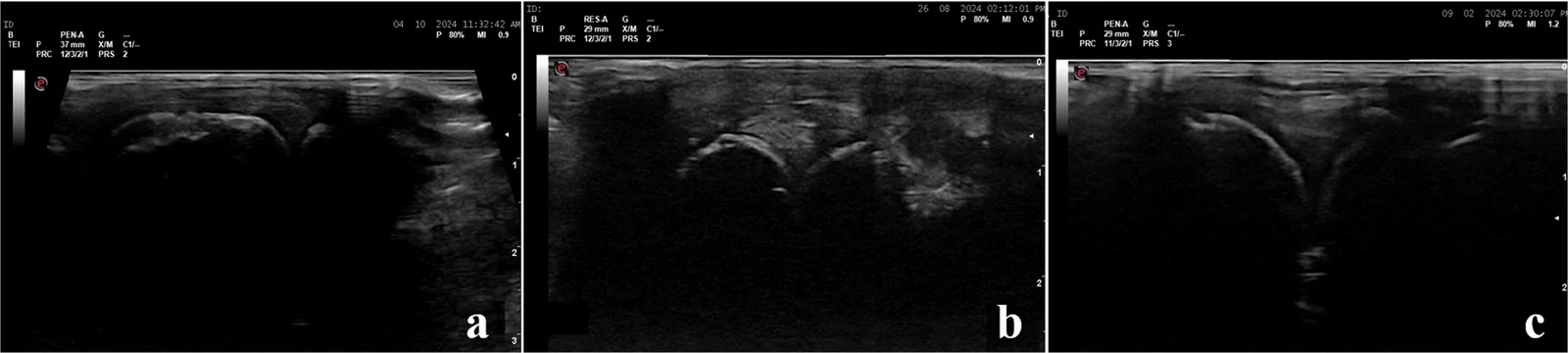
Ultrasonography assessment of articular cartilage in the stifle joint of dogs. Longitudinal view via a medial approach capturing the femoral condyle, medial meniscus, and tibial articular surface. Examples show normal-thickness articular cartilage (**a**), thin articular cartilage (**b**) and thickened articular cartilage (**c**)

A single evaluator with expertise in this field performed stifle ultrasound at preoperative and postoperative time points (3) through (7), following previous protocols (Arnault et al. 2009; Mahn et al. 2005). The medial, lateral, and cranial surfaces of the stifle were assessed, including the evaluation of the medial meniscus using the patellar and collateral ligaments as reference points. The analysis was conducted to grade articular effusion in the recess between the femoral condyle and infrapatellar region on the medial and lateral aspects of the joint as absent, mild, moderate, or severe (Fig. 1) and to evaluate the articular cartilage, classifying it as normal thickness, thin, or thick/swollen based on the contact region of the femoral condyle and medial meniscus (Fig. 2; Ramírez-Flores et al. 2017). Preoperative examinations were also performed to identify meniscal tears.

### Statistical analysis

The clinical, ultrasound, and radiographic data from pre- and post-operative evaluations of each patient were quantified and compiled in a spreadsheet. Qualitative data were analyzed discursively and compared with the available literature and expected results. Spearman’s correlation analysis was performed to compare the results among patients. Quantitative data and dependent variables were analyzed using the mode, mean, and median, with standard deviations calculated for each parameter. Ordinal (scale) data were expressed as median and ranges in tables and analyzed using non-parametric tests. Between-group comparisons at each time point were performed using the Mann–Whitney U test, and within-group comparisons over time were assessed using Friedman’s repeated measures test, followed by post hoc pairwise comparisons when appropriate. Change-from-baseline (Δ) values were calculated for key clinical outcomes (lameness grade, stifle pain, and radiographic OA) by subtracting baseline (preoperative) scores from each postoperative time point. This approach allowed each subject to serve as its own reference and helped account for baseline variability between groups. Due to the ordinal nature of the data and the limited sample size, Δ values were compared between groups at each time point using the Mann–Whitney U test.

Continuous variables were assessed for normality using the Shapiro–Wilk test. Variables meeting normality assumptions were expressed as mean ± standard deviation and analyzed using parametric tests. Consolidation time between groups was compared using Student’s t-test. Accelerometry data were analyzed within each group over time using a one-way repeated measures analysis of variance (ANOVA); when significant, the Tukey test was employed for multiple comparisons of means. Furthermore, within each group, the mean time spent at each activity level was compared using ANOVA, with the Tukey test used for pairwise mean comparisons in variables with statistically significant differences. In all analyses, the software SigmaStat 3.5 for windows (Systat Software Inc., Chicago, IL, EUA) was adopted, and a 95% confidence interval (*P* ≤ 0.05) was set.

## Results

### Patients’ data

Of the 20 patients included, one was excluded at the owner’s discretion, resulting in a total of 19 patients completing all evaluations. The CG comprised nine dogs: four mixed breed (44.44%), two Labradors (22.22%), and one each of Cane Corso, Boxer, and American Bully (11.11% each), with a mean age of 5.1 ± 1.9 years and a body weight of 26.4 ± 8.8 kg. The ADSC-Se group consisted of 10 dogs, predominantly mixed breed (*n* = 5, 50%), followed by one each of English Bulldog, Shih Tzu, Shar-Pei, American Pit Bull Terrier, and Belgian Shepherd (10% each), with a mean age of 6.5 ± 3.6 years and a body weight of 23.5 ± 7.7 kg. No differences in age or body weight were observed between groups (*P* > 0.05).

The mean duration from the onset of clinical signs to treatment was 44.9 ± 48.1 and 35.7 ± 43.4 days for the CG and ADSC-Se group, respectively (*P* > 0.05). The preoperative TPA was 28.8° ± 4.9° and 26.4° ± 3.4° in the CG and ADSC-Se group, respectively (*P* > 0.05).

### Surgical outcomes

Intraoperative complications occurred in three patients (15.79%), with two in the CG (2/9, 22.22%) and one in the ADSC-Se (1/10, 10%). These complications included vascular branch injury of the cranial tibial artery (*n* = 2) and partial iatrogenic injury to the patellar ligament (*n* = 1).

**Fig. 3:**
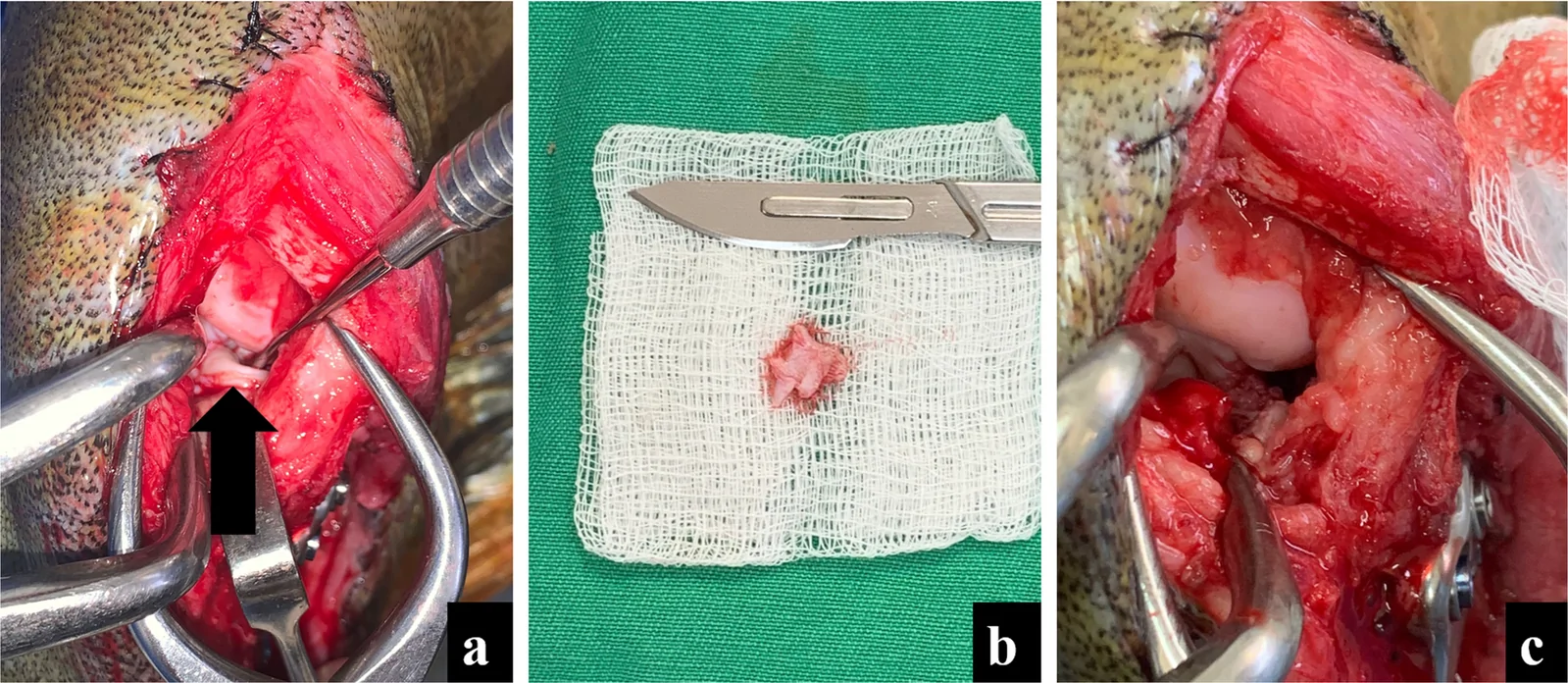
Intraoperative assessment of the stifle after tibial plateau leveling osteotomy in dogs. Medial parapatellar arthrotomy with joint distraction, demonstrating a medial meniscus lesion characterized by caudal pole eversion (arrow) (**a**). Occurrence of the medial meniscus caudal pole following partial meniscectomy (**b**). Intra-articular view of the stifle joint post-meniscectomy (**c**)

Intraoperatively, macroscopic analysis following arthrotomy was performed to analyze the degree of DJD and the integrity of the medial meniscus. In the CG, mild DJD was most prevalent (*n* = 4, 44.44%), followed by the absence of DJD (*n* = 3, 33.33%) and moderate DJD (*n* = 2, 22.22%). In the ADSC-Se, mild DJD was also predominant (*n* = 6, 60%), followed by moderate (*n* = 2, 20%), severe (*n* = 1, 10%), and absent DJD (*n* = 1, 10%). Macroscopic DJD severity differed significantly between groups (*P* = 0.0057). Medial meniscal injuries were identified in nine patients (47.37%), including three in the CG (3/9, 33.33%) and six in the ADSC-Se (6/10, 60%). All injuries necessitated partial meniscectomy (*n* = 9, 47.37%; Fig. 3). Caudal pole eversion (*n* = 7), bucket-handle tear (*n* = 1), and radial fissure (*n* = 1) were identified as meniscal injuries. The postoperative TPA measurements were 5.0° ± 3.4° and 4.8° ± 1.9° for the CG and ADSC-Se group, respectively.

Postsurgical complications were observed in both groups. In the CG group, two patients experienced minor complications: one presented a pivot shift that spontaneously resolved after 90 days post-TPLO and one developed postsurgical uremia after one week of surgery. In the ADSC-Se group, three dogs experienced minor complications: one developed a surgical site infection with osteomyelitis approximately 60 days after surgery, one experienced uremia 10 days after procedure, and one exhibited inflammation secondary to insect bites on the operated limb after 30 days of TPLO. Consequently, one patient in the CG group and two in the ADSC-Se group required an extension of the therapeutic protocol and required pain management for 60 days.

### Accelerometry assessment

Accelerometry analysis revealed no statistically significant differences in the patients’ activity levels within each group at different time points, demonstrating similar pre- and post-surgical activities (Table 1). Similarly, the mean activity levels showed no differences between the CG and ADSC-Se group at various time points (Table 2). However, when considering vigorous activity, a statistical difference emerged. For both the CG and ADSC-Se group, vigorous activity was reduced at the 24-h postsurgical time point, with a significant increase observed in this category at the 30- and 60-day periods relative to the immediate postoperative period (Table 1). Although differences were not statistically significantly detected regarding the time spent in each activity category, the ADSC-Se group demonstrated longer durations of vigorous activity compared with the CG at time points (1), (2), and (4) through (6). Furthermore, at time point (4), a critical period in recovery, no significant differences were found in the patient’s activity between the groups with respect to meniscal tears, macroscopical DJD severity, perioperative complications, clinical lameness grade, or stifle pain grade (*P* ≥ 0.05).

**Table 1:** Accelerometry-based activity levels, expressed in minutes for each category over 24 h, for the control and the adipose-derived stem cell-enriched secretome (ADSC-Se) groups at different time points

Time points	Sedentary	Moderate	Vigorous	*P*	
Control group					
Preoperative	1318.4 ± 49.1^A^	110.3 ± 46.7^B^	11.3 ± 4.5^abC^	< 0.001	
(1)	1322.1 ± 103.4^A^	113.7 ± 99.4^B^	4.2 ± 4.2^bC^	< 0.001	
(2)	1283.4 ± 143.1^A^	150.0 ± 142.0^B^	6.5 ± 3.1^abC^	< 0.001	
(3)	1299.4 ± 42.5^A^	127.2 ± 39.5^B^	13.4 ± 11.9^abC^	< 0.001	
(4)	1270.3 ± 56.1^A^	154.8 ± 48.6^B^	14.9 ± 9.6^aC^	< 0.001	
(5)	1285.5 ± 80.2^A^	139.9 ± 69.7^B^	14.6 ± 11.0^aC^	< 0.001	
(6)	1322.2 ± 39.2^A^	107.9 ± 35.3^B^	9.9 ± 4.9^abC^	< 0.001	
(7)	1243.2 ± 122.0^A^	142.7 ± 78.7^B^	16.2 ± 15.5^abC^	< 0.001	
P	0.282	0.743	0.039		
ADSC-Se group					
Preoperative	1297.6 ± 62.4^A^	130.5 ± 59.2^B^	12.0 ± 5.7^abcC^	< 0.001	
(1)	1328.8 ± 43.3^A^	105.8 ± 39.3^B^	5.4 ± 4.7^cC^	< 0.001	
(2)	1324.7 ± 38.0^A^	107.2 ± 34.2^B^	8.1 ± 5.2^bcC^	< 0.001	
(3)	1444.7 ± 405.0^A^	127.7 ± 56.2^B^	11.6 ± 10.3^abcC^	< 0.001	
(4)	1439.5 ± 407.9^A^	128.9 ± 62.2^B^	15.6 ± 10.8^abC^	< 0.001	
(5)	1291.1 ± 48.7^A^	132.8 ± 41.3^B^	16.1 ± 10.1^aC^	< 0.001	
(6)	1277.8 ± 53.9^A^	148.0 ± 47.9^B^	14.3 ± 7.7^abcC^	< 0.001	
(7)	1299.1 ± 52.6^A^	126.1 ± 44.7^B^	14.8 ± 13.0^abcC^	< 0.001	
P	0.324	0.185	0.025		

This means sharing the same superscript letter—lowercase in the column and uppercase in the row—did not differ significantly based on the Tukey test (*P* > 0.05)

**Table 2:** Comparison of accelerometry-based activity levels, expressed as mean minutes for each category over 24 h, between the control and the adipose-derived stem cell-enriched secretome (ADSC-Se) groups, at different time points

	Sedentary	Moderate	Vigorous	*P*
Preoperative				
Control	1318.4 ± 49.1^A^	110.3 ± 46.7^B^	11.3 ± 4.5^C^	< 0.001
ADSC-Se	1297.6 ± 62.4^A^	130.5 ± 59.2^B^	12.0 ± 5.7^C^	< 0.001
P	0.433	0.424	0.771	
Time point (1)				
Control	1322.1 ± 103.4^A^	113.7 ± 99.4^B^	4.2 ± 4.2^C^	< 0.001
ADSC-Se	1328.8 ± 43.3^A^	105.8 ± 39.3^B^	5.4 ± 4.7^C^	< 0.001
P	0.855	0.820	0.554	
Time point (2)				
Control	1283.4 ± 143.1^A^	150.0 ± 142.0^B^	6.5 ± 3.1^C^	< 0.001
ADSC-Se	1324.7 ± 38.0^A^	107.2 ± 34.2^B^	8.1 ± 5.2^C^	< 0.001
P	0.391	0.367	0.437	
Time point (3)				
Control	1299.4 ± 42.5^A^	127.2 ± 39.1^B^	13.4 ± 11.9^C^	< 0.001
ADSC-Se	1444.7 ± 405.0^A^	127.6 ± 56.2^B^	11.6 ± 10.3^C^	< 0.001
P	0.300	0.984	0.734	
Time point (4)				
Control	1270.3 ± 56.0^A^	154.8 ± 48.6^B^	14.9 ± 9.6^C^	< 0.001
ADSC-Se	1439.5 ± 407.9^A^	128.9 ± 62.2^B^	15.6 ± 10.8^C^	< 0.001
P	0.235	0.330	0.887	
Time point (5)				
Control	1285.5 ± 80.2^A^	139.9 ± 69.7^B^	14.6 ± 11.0^C^	< 0.001
ADSC-Se	1291.1 ± 48.7^A^	132.8 ± 41.3^B^	16.1 ± 10.1^C^	< 0.001
P	0.854	0.788	0.767	
Time point (6)				
Control	1322.2 ± 39.2^A^	107.9 ± 35.3^B^	9.9 ± 4.9^C^	< 0.001
ADSC-Se	1277.8 ± 53.9^A^	148.0 ± 47.8^B^	14.2 ± 7.7^C^	< 0.001
P	0.058	0.055	0.168	
Time point (7)				
Control	1243.2 ± 122.0^A^	142.7 ± 78.7^B^	16.2 ± 15.5^C^	< 0.001
ADSC-Se	1299.1 ± 52.6^A^	126.0 ± 44.7^B^	14.8 ± 13.0^C^	< 0.001
P	0.204	0.572	0.841	

Means accompanied by different superscript capital letters in the row indicate statistically significant differences according to Tukey’s test (*P* < 0.05).

### Lameness assessment

The clinical gait assessment for the CG group revealed that severe lameness (grade 3) was the most common preoperative grade (*n* = 6, 66.66%) and at the 24-h time point (*n* = 4, 44.44%), with a statistically significant reduction in lameness observed only after 60 days postoperatively compared with preoperatively (Table 3). For the ADSC-Se group, although patients were predominantly distributed among lameness grades 2, 3 and 4 preoperatively (*n* = 3, 30% for each grade ), a predominance of functional loss (grade 4; *n* = 5, 50%) was observed at 24 h postoperatively. Nonetheless, a significant decrease in the lameness grade was observed at 15 days postoperatively relative to the preoperative grade (Table 3).

**Table 3:** Median and range (minimum and maximum) of lameness grades in the control and the adipose-derived stem cell-enriched secretome (ADSC-Se) groups, at different time points

Time points	Control	ADSC-Se	*P*
Preoperative	3 (1–3)^ab^	3 (1–4)^a^	0.486
(1)	3 (2–4)^a^	3 (1–4)^a^	0.640
(2)	2 (1–3)^ab^	2 (1–4)^ab^	0.357
(3)	1 (0–4)^abc^	1 (0–3)^bc^	0.797
(4)	0 (0–3)^bc^	0 (0–3)^c^	0.858
(5)	0 (0–1)^c^	0 (0–1)^cd^	0.651
(6)	0 (0–0)^c^	0 (0–0)^d^	1.000
(7)	0 (0–0)^c^	0 (0–1)^d^	0.399
P	< 0.001	< 0.001	

Medians with the same lowercase superscript letter in the column did not differ significantly according to Tukey’s test (*P* > 0.05)

The Δ analysis for lameness scores demonstrated a greater magnitude of improvement in the ADSC-Se group compared to the CG during the early postoperative period. At 15 days, median Δ values were − 1.5 and − 1 for the ADSC-Se and CG, respectively (*P* = 0.31). At 30 days, the difference was more pronounced (median Δ: -3 vs. -2, respectively; *P* = 0.18). Although these differences did not reach statistical significance, a consistent trend toward faster clinical improvement was observed in the ADSC-Se group.

### Stifle pain assessment

Five (*n* = 5 upon 9, 55,55%) control dogs exhibited mild pain (score 1) from the preoperative period through 60 days postoperatively, with a significant reduction relative to the preoperative period observed only at 90 days postoperatively (Table 4). Conversely, the ADSC-Se group experienced predominantly moderate pain in the preoperative period (*n* = 7, 70%), which transitioned to an absence of pain at subsequent time points, showing a significant decrease in pain scores after 15 days post-TPLO (Table 4). Although pain levels were significantly higher in the ADSC-Se group than in the CG in the preoperative period, it reduced pain scores to levels comparable to those of the CG within the first 15 days postoperatively, with statistically superior outcomes observed at the time point (5).

**Table 4:** Median and range (minimum and maximum) of stifle pain grades in the control and the adipose-derived stem cell-enriched secretome (ADSC-Se) groups, at different time points

Time points	Control	ADSC-Se	*P*
Preoperative	1 (1–3)^a^	2 (2–3)^a^	0.022
(3)	1 (0–2)^ab^	0 (0–1)^b^	0.112
(4)	1 (0–1)^ab^	0 (0–3)^b^	0.210
(5)	1 (0–1)^ab^	0 (0–0)^b^	0.009
(6)	0 (0–1)^b^	0 (0–0)^b^	0.343
(7)	0 (0–1)^b^	0 (0–1)^b^	1.000
P	< 0.001	< 0.001	

Medians with the same lowercase superscript letter in the column did not differ significantly according to Tukey’s test (*P* > 0.05)

The Δ analysis for stifle pain scores demonstrated a significantly greater reduction in the ADSC-Se group compared to the CG during the early postoperative period. At 15 days, median Δ values were − 2 and − 1 for the ADSC-Se and CG, respectively (*P* = 0.04). A similar pattern was observed at 30 days (median Δ: -2 vs. -1, respectively; *P* = 0.03). At later time points, both groups showed minimal pain scores, reducing differences between treatments.

### Radiographic assessment

The radiographic evaluation demonstrated that complete bone healing occurred after 86.7 ± 18.0 and 81.0 ± 20.2 days for CG and ADSC-Se group, respectively. Although ADSC-Se required fewer days to achieve complete healing, no statistically significant difference was found between the groups. However, at 30 days postoperatively, when assessed using the 5- and 10-point grading scales for bone healing, the ADSC-Se group revealed a more advanced ossification process compared with CG (Table 5).

**Table 5:** Radiographic bone healing assessment in the control and the adipose-derived stem cell-enriched secretome (ADSC-Se) groups using 5- and 10-point scales at time point (4)

Bone healing scales^a^	Control	ADSC-Se	*P*
10 points	2.9 ± 1.8^B^	4.8 ± 1.8^A^	0.036*
5 points	1.6 ± 0.7^B^	2.6 ± 1.0^A^	0.017*

^a^According to Moore et al. (2019); ^*^Statistical difference (*p* < 0.05)

**Fig. 4:**
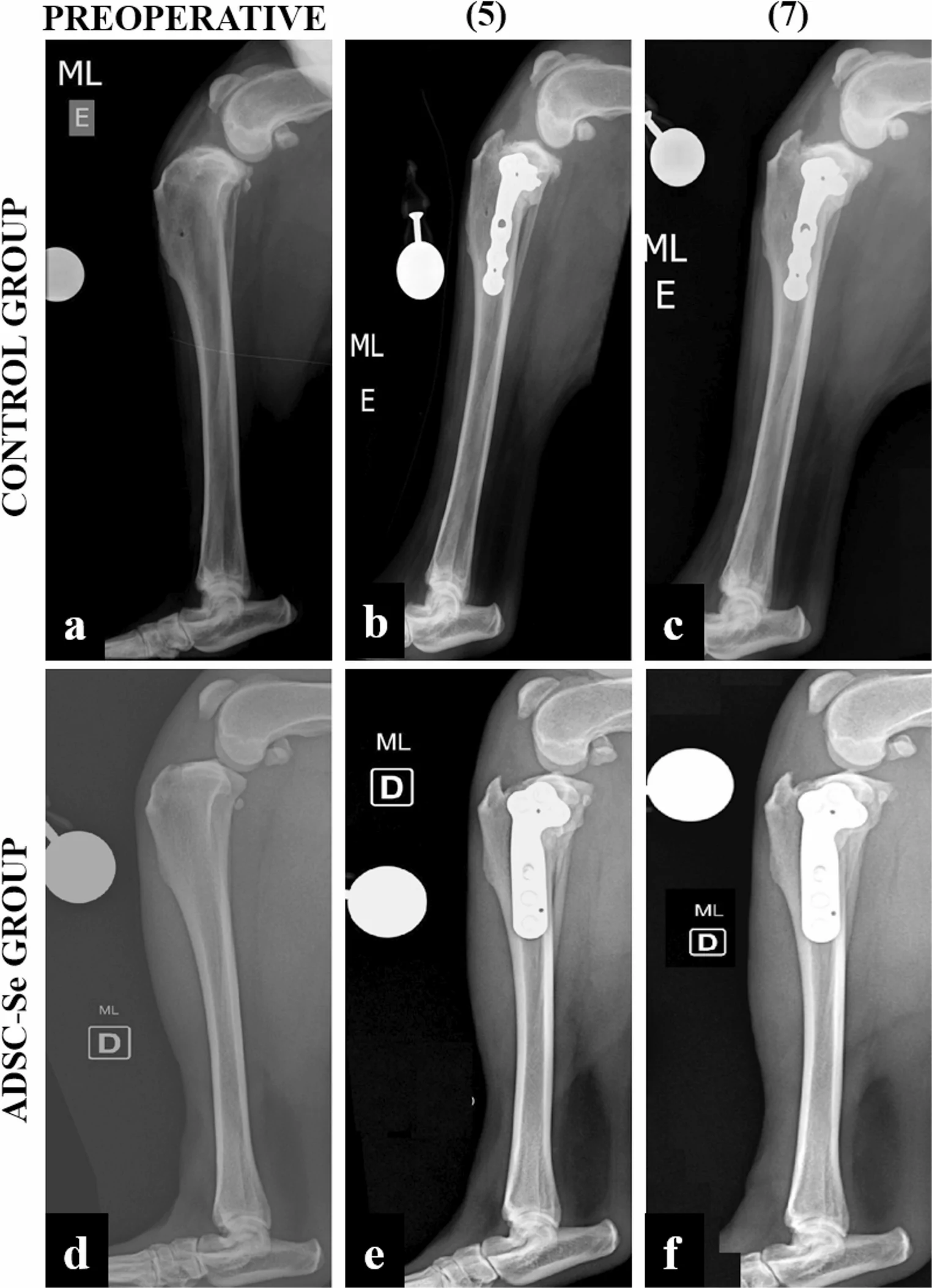
Sequential radiographic assessment of osteoarthritis progression in the control (**a**-**c**) and the adipose-derived stem cell-enriched secretome (ADSC-Se) groups (**d**-**f**). The first column (**a** and **d**) displays the preoperative time point, followed by images acquired at 60 ± 5 days (time point 5, **b** and **e**) and 120–150 days (time point 7, **c** and **f**) postoperatively

Based on the radiographic grading of stifle OA (Fig. 4), the CG exhibited a progressive worsening of OA across the evaluated time points, especially at time point (7) in relation to the preoperative moment and time point (5) compared with the immediate postoperative (Table 6). In the ADSC-Se group, the OA score decreased at time point (3) relative to the preoperative assessment, and subsequent evaluations demonstrated that the OA grade remained stable at all postoperative time points, revealing no progression of OA (Table 6). When comparing the groups, the median OA scores were higher in the CG than in the ADSC-Se group from time points (3) to (7) (Table 6).

**Table 6:** Median and range (maximum and minimum) of radiographic osteoarthritis progression on a six-point scale (Jeong et al. 2021) in the control and the adipose-derived stem cell-enriched secretome (ADSC-Se) groups

Time point	Control	ADSC-Se	*P*
Preoperative	1 (0–3)^bc^	1 (0–2)^a^	0.169
Immediate postoperative	0 (0–3)^c^	0 (0–2)^ab^	0.394
(3)	0 (0–3)^c^	0 (0–2)^b^	0.228
(4)	2 (0–3)^bc^	0 (0–2)^b^	0.036
(5)	2 (0–3)^ab^	0 (0–2)^b^	0.004
(6)	2 (0–4)^ab^	0 (0–3)^b^	0.003
(7)	2 (2–4)^a^	0 (0–3)^b^	< 0.001
P	< 0.001	< 0,001	

Medians sharing the same superscript letter—lowercase in the column and uppercase in the row—did not significantly differ according to the Tukey test (*P* > 0.05)

The Δ analysis for radiographic OA scores suggested a tendency toward reduced OA progression in the ADSC-Se group compared to the CG. At 30 days, the control group showed a median increase (Δ = +1), whereas the treated group remained stable (Δ = 0; *P* = 0.08). A similar trend was observed at 60 days (*P* = 0.07). Although these differences did not reach statistical significance, they suggest a potential protective effect against OA progression.

### Ultrasonographic assessment

Preoperative articular ultrasonography was used to assess ligamentous injuries and meniscal integrity. All patients (*n* = 19, 100%) in both groups were diagnosed with ligamentous disease characterized by total or partial rupture or fiber degeneration. Four patients (44.44%) in the CG were diagnosed with meniscal tears, whereas this condition was observed in 7 patients (70%) in ADSC-Se group. Ultrasound findings were compared with the macroscopic examination. The ultrasonographic assessment effectively identified meniscal integrity or tears in six patients from the CG (66.7%) and seven from the ADSC-Se group (70%). Considering both groups, the sensitivity, specificity, and accuracy for the meniscal evaluation were 77.77%, 60%, and 68.42%, respectively.

Articular effusion and articular cartilage characteristics are ultrasound parameters used for OS assessment over time. In the preoperative period, both groups predominantly had mild articular effusion (*n* = 8, 88.88% in CG; *n* = 6, 60% in ADSC-Se). In CG, effusion persisted from time point (3) to (6), with mild and moderate effusion occurring at equal frequencies (*n* = 4, 44.44% each), demonstrating no reduction throughout the evaluation period (Table 7). In ADSC-Se, mild effusion was predominant at time point (3) (*n* = 8, 80%). However, from time points (4) to (7), effusion was largely absent, indicating a significant reduction from 30 days postoperatively compared with the preoperative period (Table 7). Furthermore, effusion was statistically significantly different between the two groups at all-time points.

**Table 7:** Median and range (maximum and minimum) of ultrasonographic assessment of articular effusion intensity in the control and the adipose-derived stem cell-enriched secretome (ADSC-Se) groups, at different time points

Time point	Control	ADSC-Se	*P*
Preoperative	1 (1–2)	1 (1–2)^a^	0.181
(3)	2 (1–3)	1 (0–1)^ab^	0.007
(4)	2 (1–3)	0 (0–1)^b^	< 0.001
(5)	2 (1–3)	0 (0–1)^b^	< 0.001
(6)	2 (1–3)	0 (0–0)^b^	< 0.001
(7)	1 (0–2)	0 (0–1)^b^	0.002
P	0.068	< 0.001	

0, absence; 1, mild; 2, moderate; 3, severe. Medians with the same lowercase superscript letter in the column did not differ significantly according to Tukey’s test (*P* > 0.05)

Ultrasonographic evaluation of the articular cartilage revealed a predominance of normal thickness in the CG (*n* = 5, 55.55%) and swollen cartilage in the ADSC-Se group (*n* = 4, 40%) during the preoperative period. From 15 to 60 days postoperatively, the number of patients with swollen cartilage was highest (*n* = 8, 88.88% at each time point), with a significant increase up to time point (5). Cartilage parameters returned to preoperative levels only after 120 days following TPLO (Table 8). Conversely, patients in the ADSC-Se group exhibited normal cartilage thickness at all postoperative time points, demonstrating a significant reduction in this inflammatory parameter from 30 days postoperatively until the end of the analysis (Table 8). When comparing both groups, a reduction in articular cartilage damage was observed in the ADSC-Se group from 15 to 90 days after TPLO (Table 8).

**Table 8:** Median and range (maximum and minimum) of ultrasonographic assessment of articular cartilage characteristics in the control and the adipose-derived stem cell-enriched secretome (ADSC-Se) groups, at different time points

Time point	Control	ADSC-Se	*P*
Preoperative	0 (0–2)^b^	1 (0–2)^a^	0.456
(3)	2 (1–2)^a^	0 (0–2)^ab^	0.003
(4)	2 (1–2)^a^	0 (0–0)^b^	< 0.001
(5)	2 (1–2)^a^	0 (0–0)^b^	< 0.001
(6)	1 (0–2)^ab^	0 (0–0)^b^	< 0.001
(7)	0 (0–2)^b^	0 (0–1)^b^	0.081
P	0.001	< 0.001	

0, normal thickness; 1, thin; 2, thick/swollen. Medians with the same lowercase superscript letter in the column did not differ significantly according to Tukey’s test (*P* > 0.05)

## Discussion

This pilot study evaluated the intra-articular injection of the allogeneic ADSC-enriched secretome in the stifle joints of dogs with CCLD that underwent TPLO, a field that has been largely unexplored in veterinary medicine. CCLD treatment aims to promote stifle stabilization and reduce the long-term OA progression caused by the unstable joint environment (Spinella et al. 2021). Despite its widespread use, TPLO alone does not prevent postoperative OA progression (Shimada et al. 2020). This study’s findings, based on radiographic, ultrasonographic, gait, and pain assessments, suggest the potential effectiveness of ADSC-Se in reducing OA progression and accelerating recovery of TPLO-patients in the mild- and mid-term outcomes, supporting our hypothesis.

The radiographic assessment evaluated the joint inflammation patterns using a six-point scale (Moore et al. 2020). OA scores in CG progressed throughout the evaluation period, whereas the ADSC-Se group maintained consistent OA scores postoperatively. Osteophytosis progression, a key aspect of the radiographic evaluations, indicates the advancement of DJD and a worse long-term prognosis (Engdahl et al. 2023). Therefore, preventing DJD progression can be considered a positive aspect of ADSC-Se therapy, supporting previous findings (Song et al. 2021) and aligning with the authors’ hypothesis. The radiographic bone healing process was assessed during the evaluation process. Both groups required a similar time for complete bone healing; however, the ADSC-Se group exhibited advanced ossification by 30 days postoperatively. Intra-articular administration of allogeneic canine ADSC in patients with TPLO has been shown to reduce the time required for bone healing, which can be related to the cellular migration ability (Pennasilico et al. 2023). However, the secretome derived from these cells injected in the context of TPLO osteotomy healing, remains unexplored, as it exhibits only local activity (Gowen et al. 2020). The results presented here indicate bone healing process acceleration in the short term, although the time required for complete radiographic ossification was not affected. The observed effect on bone healing may be linked to earlier weight-bearing, which accelerates recovery (Monk et al. 2006).

The ultrasonographic assessment showed positive outcomes. CCLD is known to cause mechanical joint damage and alterations secondary to cranial tibial instability, resulting in both intra- and extra-articular injuries (Pennasilico et al. 2024). However, ultrasound used to assess stifle OA parameters in patients with TPLO remains an unexplored area in veterinary medicine, and postoperative intra-articular assessments at multiple time points are still limited in both clinical and research settings. Articular effusion and cartilage characteristics serve as key indicators of OA assessment through ultrasonography (Ramírez-Flores et al. 2017). Articular effusion is one of the primary alterations following CCLD, with a peak incidence of around 8 weeks post-injury and spontaneous regression by 26 weeks (D’Anjou et al. 2008). Although long-term evaluations were not performed, both groups exhibited effusion preoperatively. In CG, high effusion levels persisted in the postoperative period, highlighting an ongoing OA despite TPLO (Shimada et al. 2020). Conversely, this parameter was reduced in the ADSC-Se group, particularly at 30 days postoperatively. The anti-inflammatory effect of the secretome may be a key factor in these results, particularly associated with apoptosis regulation through Forkhead box proteins and enhanced interleukin-10 secretion (Li et al. 2022).

The stifle articular cartilage is affected in all patients with CCLD and plays a crucial role in OA pathophysiology (Agnello et al. 2021). In case of acute damage, cartilage swelling occurs, whereas chronic lesions result in thinning, erosion, and osteophytosis (Glyn-Jones et al. 2015; Kuyinu et al. 2016; Marshall et al. 2009). Research and the development of alternative therapies are essential to improve the outcomes of patients with CCLD. In this study, cartilage swelling was assessed based on previously established ultrasonographic criteria for stifle OA (Ramírez-Flores et al. 2017). Furthermore, cartilage thinning was incorporated into the methodology to account for group heterogeneity, evaluation time points, and variations in CCLD duration. In CG, cartilage swelling remained predominant until 60 days post-TPLO, a finding consistent with acute joint injuries and that of previous studies (Ramírez-Flores et al. 2017). Conversely, the cartilage thickness was found to be normal in the ADSC-Se group during the same period. These results may be attributed to ADSC-Se’s ability to reduce joint inflammation and stimulate local mesenchymal cartilage cells for differentiation (Carrera et al. 2023). Accordingly, positive outcomes were also observed in the gait analysis and stifle pain assessment.

A positive and early clinical recovery was observed in patients treated with ADSC-Se compared with CG, as evidenced by improved stifle pain and gait analysis parameters. Effective pain management immediately after TPLO is crucial and challenging, primarily due to surgical manipulation, which can result in delayed limb function recovery (Soto et al. 2014). Therefore, enhanced analgesic and recovery methods are both warranted and necessary. In this study, the reduction in stifle pain was more rapid in the ADSC-Se group than the CG group, with a statistically significant decrease observed as early as 15 days postoperatively. This outcome may be directly attributed to the anti-inflammatory and immunomodulatory properties of ADSCs and their secreted products (Sasaki et al. 2019). Moreover, OA is expected to reduce weight bearing on the affected limb, resulting in varying degrees of lameness (Wemmers et al. 2022), a condition found to be more pronounced in the CG group than in the ADSC-Se group. Patients treated with ADSC-Se showed a significant reduction in lameness as early as 15 days postoperatively, indicating superior outcomes for the combined TPLO plus ADSC-Se treatment compared with TPLO alone.

Despite the positive outcomes in managing stifle pain and gait analysis for the ADSC-Se group, the activity level assessed through accelerometry did not significantly differ between the groups. These findings were consistent with those of previous studies (Schuster et al. 2023), which reported no significant differences in activity levels before and after surgical treatment of CCLD, even when using techniques other than TPLO. Thus, clinical recovery is not statistically correlated with activity levels in patients with CCLD (Schuster et al. 2023), which justifies the inclusion of additional parameters in this study’s methodology. Although no statistically significant difference was observed between the groups, patients in the ADSC-Se group exhibited a higher mean duration of vigorous activity than those in the CG. This observation, together with the results from other assessments, should be considered when deciding on the adoption of the proposed therapy for patients with TPLO.

Given that this study was conducted with client-owned dogs from a teaching hospital, group heterogeneity may limit the results. Specifically, the ADSC-Se group included more patients than the CG group, and the breeds were not consistent between the groups. Furthermore, the clinical presentation and outcomes could significantly be influenced by the higher prevalence of meniscal tears and the intensity of macroscopic DJD in the ADSC-Se group. Another limitation of our study is that secretome was standardized on protein concentration only. We cannot estimate the average number of cells used to prepare a single dose, and we did not characterize bioactive components. This raises questions about reproducibility and hinders understanding mechanisms of action of ADSC-Se. Therefore, future prospective clinical trials with larger sample sizes, the implementation of novel objective evaluation methods, more homogeneous groups and improved characterization of the secretome are encouraged to further assess the efficacy of ADSC-Se as an adjuvant in this OA model.

ADSCs and their culture by-products represent a potential alternative therapy for OA. Initially, the direct effects of ADSCs, through articular regeneration, constituted the primary mechanism of action. In fact, the indirect effects of ADSCs are now recognized predominantly, as they reduce inflammation and create a more favorable joint environment (Sasaki et al. 2019). These effects are primarily mediated by secreted products (Carrera et al. 2023), of which were directly administered intra-articularly in this study. Using the whole secretome instead of isolated extracellular vesicles simplifies extraction and reduces costs (Bousnaki et al. 2020; Gowen et al. 2020; Tieu et al. 2020), making clinical application more feasible; however, further detailed studies are warranted to refine the assessment of its clinical safety. Patients treated with enriched secretome demonstrated potential positive clinical and imaging outcomes, particularly regarding reduced inflammatory parameters associated with stifle joint OA. These results may be attributed to the ADSC-Se’s capacity to reduce proinflammatory cytokines, inhibit macrophage activation, and decrease nitric oxide production in the articular fluid (Engdahl et al. 2023).

Our findings highlight that intra-articular injection of canine allogeneic ADSC-enriched secretome is a promising alternative therapy and suggest that the compound promotes faster clinical recovery and reduces both clinical and imaging parameters of OA in dogs with CCLD undergoing TPLO. This paper reinforces the therapeutic potential of ADSC-enriched secretome in canine OA, highlighting its suggested use as an adjuvant therapy for canine surgical treatment. However, larger clinical trials are encouraged to confirm the results.

## Supplementary Information

Below is the link to the electronic supplementary material.

Supplementary Material 1 (PDF 3.86 MB)

## Data Availability

The data that support the findings of this study are openly available in UNESP Institutional repository at (https:/hdl.handle.net/11449/295522) .
