# Supplementary material for: Intra-articular injection of allogeneic canine adipose-derived stem cell enriched secretome in dogs undergoing tibial plateau leveling osteotomy: a prospective double-blind and randomized pilot trial
Source: Vet Res Commun. 2026 Jun 13;50(5):394. doi: 10.1007/s11259-026-11343-9 (PMC13264577; doi:10.1007/s11259-026-11343-9)

Intra-articular injection of allogeneic canine adipose-derived stem cell enriched secretome in dogs undergoing tibial plateau leveling osteotomy: a prospective double-blind and randomized pilot trial

Veterinary Research Communications

Alefe Luiz Caliani Carrera[1*], Bruno Watanabe Minto[1], Brenda Mendonça Alcântara[1], Daniel Alexandre Coimbra Previtali[1], Rodrigo Carvalho Souza Faustino[1], Angélica Barreto Leite Tavares[1], Danuta Pulz Doiche[1], Camila Nogueira[1], Hilana Santos Sena Brunel[2], Rinaldo Wellerson Pereira[2], Marcelo Meller Alievi[3], Luis Gustavo Gosuen Gonçalves Dias[1], Patricia Furtado Malard[2]

[1] Department of Veterinary Clinics and Surgery, School of Agricultural and Veterinary Studies (FCAV), São Paulo State University (UNESP), Jaboticabal (SP), Brazil.

[2] Postgraduate Program in Genomic Sciences and Biotechnology, Catholic University of Brasilia (UCB), Brasília (DF), Brazil

[3]Department of Animal Medicine, Faculty of Veterinary Sciences (FAVET), Federal University of Rio Grande do Sul (UFRGS), Porto Alegre (RS), Brazil.

[*] **Corresponding author:** Alefe Luiz Caliani Carrera. Department of Veterinary Clinics and Surgery, School of Agricultural and Veterinary Studies (FCAV), São Paulo State University (UNESP). Access Road Prof. Paulo Donato Castellane s/n, Jaboticabal, State of São Paulo, Brazil, Zip code:14884-900. Phone number +55 44 99900 6272. E-mail: alefe.carrera@unesp.br. ORCiD https://orcid.org/0000-0003-3558-2664

Online resource 1. Supplementary data on characterization, donor characteristics and quality control (genetic, microbiological, and viability assessments) of allogeneic adipose-derived stem cells used for secretome production.

Section I – Materials and methods for cellular characterization

Each cell batch was cryopreserved and quarantined until cleared by quality control assays. After thawing, osteogenic differentiation was induced using a specific induction medium, and mineralization was subsequently confirmed using Alizarin Red staining under light microscopy at 100× magnification. Adipogenic and chondrogenic differentiation were similarly evaluated using Oil Red O and Alcian Blue staining, respectively. Positive differentiation into all three lineages confirmed the suitability of the cells for further use. Immunophenotyping was conducted to confirm the mesenchymal profile by assessing surface markers CD29, CD44, and CD105, as well as the pluripotency-associated markers SOX2 and OCT3/4. These analyses were performed using flow cytometry and polymerase chain reaction (PCR) with reagents specific to the canine species. Sterility confirming testing included bacterial and fungal screening, Mycoplasma spp. culture, and PCR-based detection.

Cell viability was assessed for each batch after 24 h of cryopreservation. The cells were thawed and evaluated using the Trypan Blue exclusion assay with a 1:1 dilution (cell suspension:Trypan Blue). Cell counts were performed in a Neubauer chamber under light microscopy, with 200 cells evaluated at two time points: 30 minutes and 24 hours post-thawing. The batch was considered viable if ≥80% of the cells remained viable at 24 hours. The three batches achieved viability rates of 92% and 91.5%, 94.5% and 83.5%, and 92.5% and 91% at 30 minutes and 24 hours, respectively.

Section II – Donors and quality control for each batch

Donor 1 – Female dog, 1 year and 3 months, 26kg, Mixed breed.

Batch identification: 04.008.16

HEALTH ASSESSMENT

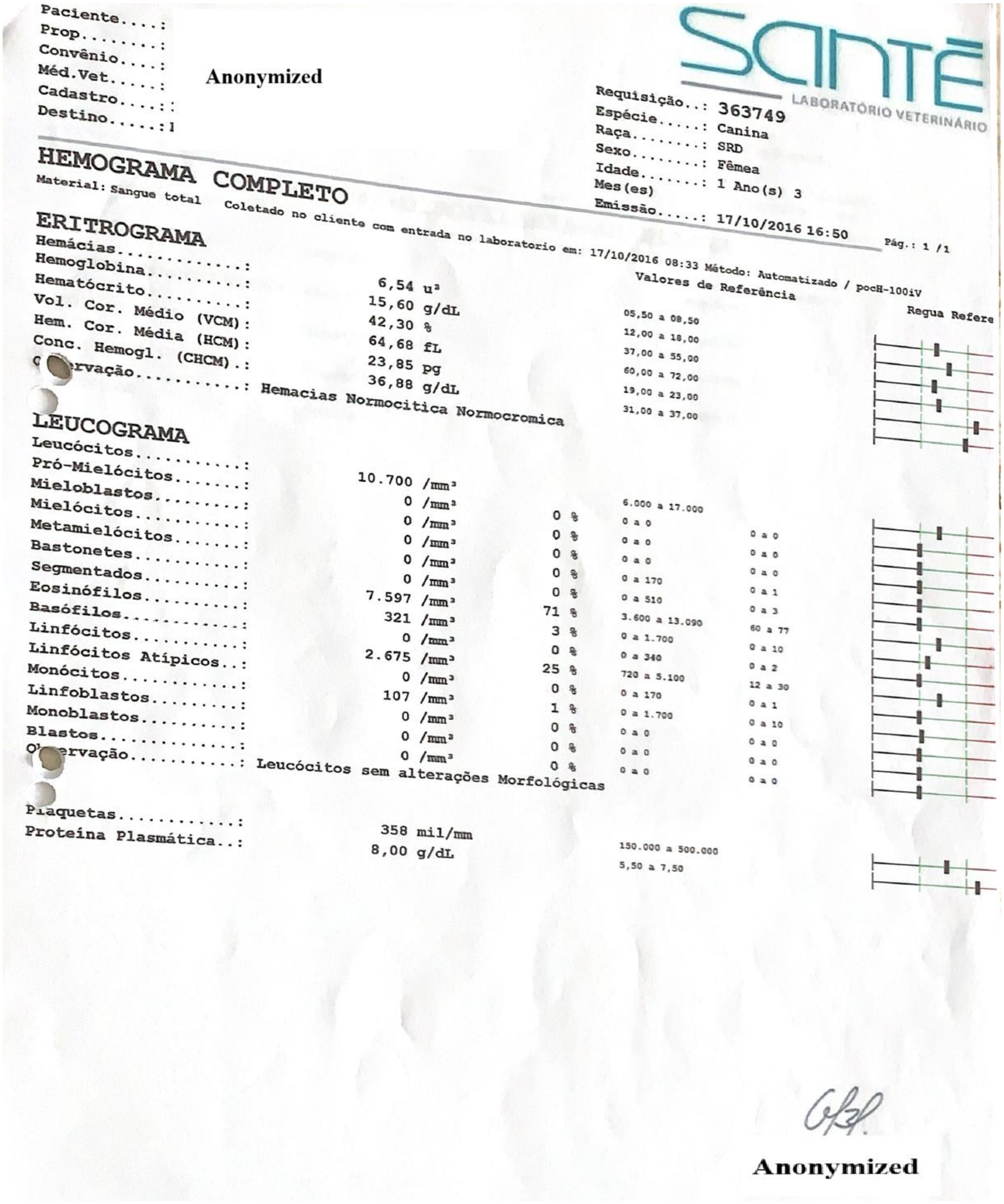

Paciente....:
Prop........:
Convênio....:
Méd.Vet.....:
Cadastro....::
Destino.....:l

Anonymized

SANTÉ LABORATÓRIO VETERINÁRIO

Requisição..: 363749
Espécie.....: Canina
Raça........: SRD
Sexo........: Fêmea
Idade.......: 1 Ano(s) 3 Mes(es)
Emissão.....: 17/10/2016 16:50

HEMOGRAMA COMPLETO

Material: Sangue total   Coletado no cliente com entrada no laboratorio em: 17/10/2016 08:33 Método: Automatizado / pocH-100iV   Pág.: 1 /1

ERITROGRAMA

		Valores de Referência
Hemácias	6,54 u³	05,50 a 08,50
Hemoglobina	15,60 g/dL	12,00 a 18,00
Hematócrito	42,30 %	37,00 a 55,00
Vol. Cor. Médio (VCM)	64,68 fL	60,00 a 72,00
Hem. Cor. Média (HCM)	23,85 pg	19,00 a 23,00
Conc. Hemogl. (CHCM)	36,88 g/dL	31,00 a 37,00

Observação..........: Hemacias Normocitica Normocromica

LEUCOGRAMA

Leucócitos	10.700 /mm³		6.000 a 17.000	
Pró-Mielócitos	0 /mm³	0 %	0 a 0	0 a 0
Mieloblastos	0 /mm³	0 %	0 a 0	0 a 0
Mielócitos	0 /mm³	0 %	0 a 0	0 a 0
Metamielócitos	0 /mm³	0 %	0 a 170	0 a 1
Bastonetes	0 /mm³	0 %	0 a 510	0 a 3
Segmentados	7.597 /mm³	71 %	3.600 a 13.090	60 a 77
Eosinófilos	321 /mm³	3 %	0 a 1.700	0 a 10
Basófilos	0 /mm³	0 %	0 a 340	0 a 2
Linfócitos	2.675 /mm³	25 %	720 a 5.100	12 a 30
Linfócitos Atípicos	0 /mm³	0 %	0 a 170	0 a 1
Monócitos	107 /mm³	1 %	0 a 1.700	0 a 10
Linfoblastos	0 /mm³	0 %	0 a 0	0 a 0
Monoblastos	0 /mm³	0 %	0 a 0	0 a 0
Blastos	0 /mm³	0 %	0 a 0	0 a 0

Observação..........: Leucócitos sem alterações Morfológicas

Plaquetas	358 mil/mm	150.000 a 500.000
Proteína Plasmática	8,00 g/dL	5,50 a 7,50

Regua Refere

Anonymized

Paciente..: [illegible]
Prop........
Convênio....
Méd.Vet.....
Cadastro....
Destino.....

Anonymized

SANTÉ LABORATORIO VETERINÁRIO

Requisição..: 363749
Espécie.....: Canina
Raça........: Não informado
Sexo........: Fêmea
Idade.......: 1 Ano(s) 3 Mes(es)
Emissão.....: 17/10/2016 17:08

Pág.: 1 /1

EHRLICHIA CANIS+LYME+DIROFILARIA+ANAPLASMA

Material: SORO Coletado no cliente com entrada no laboratorio em: 17/10/2016 08:40 Método: ELISA

		Valores de Referência
ERLYCHIA CANIS...........	: Negativo	Negativo
ANAPLASMA PHAGOCITOPHYLUM	: Negativo	Negativo
DOENÇA DE LYME...........	: Negativo	Negativo
DIROFILARIA IMMITIS......	: Negativo	Negativo
O[illegible]ERVAÇÃO...............	: Negativo	

Anonymized

53

Paciente....:
Prop........:
Convênio....:
Méd.Vet.....:
Cadastro....:
Destino.....:

Anonymized

SANTÉ
[illegible]RATÓRIO VETERINÁRIO

Anonymized

LEISHMANIOSE CANINA

Material: Soro   Coletado no cliente com entrada no laboratorio em: 17/10/2016 08:33 Método: Imunocromatográfico+Elisa

[illegible]ssão.....: 01/11/2016 18:20   Pág.: 1 /1

MÉTODO: IMUNOCROMATOGRÁFICO

PARTIDA............: 001/16 - 2104DA015
VALIDADE...........: 30/11/2017
RESULTADO..........: Não Reagente

MÉTODO: ENSAIO IMUNOENZIMÁTICO (ELISA)
[illegible] COM LICENÇA NO MINISTÉRIO DA AGRICULTURA - MAPA Nº 7.434/2000

[illegible]RTIDA............: 03/16
VALIDADE...........: 28/02/2017
RESULTADO..........: Não Reagente

INTERPRETAÇÃO.......: CASOS DE DIVERGENCIA ENTRE OS TESTES PODEM CORRESPONDER AO INICIO DA SOROCONVERSÃO, REAÇÕES CRUZADAS E/OU INESPECIFICAS, OU FALÊNCIA DO SISTEMA IMUNE. RECOMENDA-SE REALIZAR NOVO EXAME APÓS 30 DIAS DO ÚLTIMO.

NOTA................: - O EXAME ESTA SUJEITO, EMBORA RARAMENTE, A OCORRÊNCIA DE RESULTADOS FALSO-NEGATIVO E FALSO-POSITIVO, QUE É UMA CARACTERÍSTICA DE VARIAÇÕES PRÉ-ANALÍTICAS E DAS METODOLOGIAS. SUGERIMOS O ACOMPANHAMENTO MÉDICO VETERINÁRIO DA SINTOMATOLOGIA CLÍNICA.

- TÉCNICAS DIAGNÓSTICAS ADICIONAIS PODERÃO SER UTILIZADAS,COMO O ENSAIO IMUNOCROMATOGRÁFICO. ENTRETANTO,PARA EFEITO DE SAÚDE PÚBLICA SÓ SERÃO ACEITOS COMO TRIAGEM TESTE DE PLATAFORMA DUPLA K28 E TESTE ELISA PARA CONFIRMAÇÃO. AMBOS OS TESTES COM SENSIBILIDADE E ESPECIFICIDADE IGUAL OU SUPERIOR A 90%.

- AS DUAS TECNICAS SOROLÓGICAS (TR-DPP E ELISA) SÃO RECOMENDADAS PELO MINISTÉRIO DA SAÚDE PARA O DIAGNÓSTICO DE LEISHMANIOSE VISCERAL CANINA. O CÃO É CONSIDERADO UM CASO CONFIRMADO QUANDO AS DUAS TÉCNICAS APRESENTAREM RESULTADOS REAGENTES.

Fonte: Nota tecnica conjunta n. 01/2011 - CGDT-CGLAB/DEVIT/SVS/MS
Nota tecnica n. 01/2013 - SDP/DECD/IOM/FUNED
Oficio SDP n. 451/2013
Ofício SDP n. 263/2014

Exame realizado em laboratorio de apoio. O original encontra-se à disposição no Santé Laboratorio.

Anonymized

bio cell
Email: laboratorio@biocell.com.br
End: R. 25 Sul, lote 30 - Águas Claras/DF
Telefone: (61) 9921-6776

Data: 21/08/2023
Responsável Técnico: Karine R D Silveira

Identificação BIOCELL 04.008.16 LT 080121.1

Laudo de Imunofenotipagem

Método: Imunofenotipagem por citometria de fluxo
Material: Células-tronco mesenquimais

Marcador

CD90 (APC)	MARCADOR POSITIVO
CD44 (FITC)	MARCADOR POSITIVO
CD29 (PerCy-p)	MARCADOR POSITIVO
MHCII (FITC)	MARCADOR NEGATIVO

Resultados

Triple Positive	Double Negative	Single Positive	Single Positive	Single Positive	Single Positive	Single Negative
CD44/ CD29/ CD90	CD44/ CD29/ CD90	CD44	CD29	CD90	MHC II	MHC II
99,87%	0,00%	0,06%	0,00%	0,13%	0,28%	99,72%

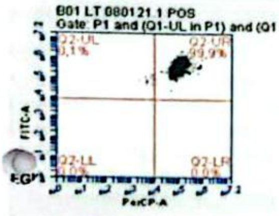

Plot FITC X PerCP-A: Marcadores CD44/CD29/CD90.

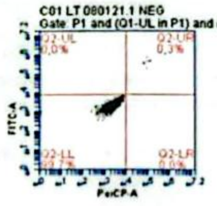

Plot FITC X PerCP-A: Marcador MHCII.

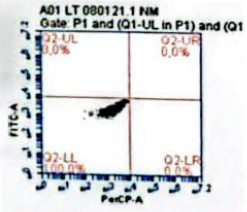

Plot FITC X PerCP-A: Sem Marcador.

Descritivo

Expressão dos marcadores positivos de células-tronco CD44, CD29 e CD90 na superficie celular em 99,87% do plot amostral analisado. Expressão dos marcadores negativos de células-tronco e MHCII em 0,28% do plot amostral analisado.

Conclusão

Em função somatória dos fatos analisados, o grupo amostral refere-se às células-tronco mesenquimais. Células se encontram aptas para uso.

Responsável Técnico<br>Data: 31/08/23	Responsável pela Garantia da Qualidade<br>Data: 01/09/2023	Responsável pelo Controle da Qualidade<br>Data: 31/08/2023

STERILITY TESTING

Relatório de Ensaio Nº **0000601931**

[illegible]a de emissão: **10/08/2024**

Identificação do Cliente

Cliente: BIO CELL TERAPIA CELULAR LTDA
Contato:
Endereço: RUA 25 SUL BLOCO A LOJA 105, LOTE 30COMPLEMENTO : CIDADE ÁGUAS CLARAS

CNPJ/CPF: 19.283.681/0001-70
Telefone:(61) 999216776
Email:

Dados da Amostra: AMOSTRA 03: BIOCELL 04.008.16 LT 190624.3

Data de Coleta: 01/08/2024
Local da Coleta: -
Responsável Pela Coleta: Cliente
Data da Fabricação: 19/06/2024
Lote: 190624.3

Data Recebimento: 02/08/2024 12:50:00
Hora Coleta: 11:00:00
Quantidade: 10 mL
Data de Validade: -
Temperatura coleta(°C): AMBIENTE
Temperatura recebimento(°C): 10.6

Informações gerais: COLETADO POR: V Anonymized
Plano de amostragem: Plano de amostrag [illegible]ssado

Resultados Analíticos

Ensaio(s)	Resultado(s)	Unidade	Valor de referência	Metodologia(s)	Data Ensaio(s)
Teste de esterilidade - IN SITU	SEM CRESCIMENTO	-	Sem crescimento	FARMACOPEIA BRASILEIRA – VOLUME 1 – MÉTODO – 5.5.3.2.1. 2019	

Norma de referência

Declaração de Conformidade

A presente amostra atende aos padrões estabelecidos pela legislação vigente conforme norma de referência citada.

Os resultados obtidos foram comparados com o valor de referência de acordo com a legislação solicitada. Os resultados com valores acima do valor de referência foram considerados resultados não conforme.
Os resultados quantitativos, quando expressos, não consideram a incerteza inerente aos métodos analíticos como regra para decisão quanto à conformidade a uma especificação ou norma.
O(s) ensaio(s) sem valor(es) de referência estabelecido devem ser avaliados tecnicamente pelo responsável da empresa.

Opiniões e Interpretações
"As opiniões e interpretações expressas abaixo não fazem parte do escopo da acreditação deste laboratório"

NOTAS

- Estes resultados se referem apenas à amostra analisada.
- Os serviços são realizados nas instalações permanentes do laboratório, exceto os descritos como "In Loco".
- Na coleta realizada pelo cliente, a amostragem é de sua responsabilidade e as amostras serão analisadas como recebidas, salvo quando as mesmas não apresentarem condições.
- É responsabilidade do cliente o preenchimento adequado na solicitação de análise, das informações referentes aos dados da amostra, incluindo data e hora de coleta, data de fabricação e validade, lote e responsável e local da amostragem.
- Este relatório somente poderá ser reproduzido em sua totalidade. O Laboratório F.A.Z Análises se isenta de qualquer responsabilidade pela reprodução parcial do mesmo.

VetDNA DIAGNÓSTICOS MOLECULARES

VetDNA Diagnósticos Moleculares
Rua La Salle, 290
CEP 18608-240 Vila Nova Botucatu
Botucatu/SP
CNPJ: 11.483.985/0001-70

Animal......: 04.008.16 LT 190624.3
Cliente.....:
Médico......:
Covênio.....: Anonymized
Entrada.....:
Proprietário:

Requisição..: 140931
99220Espécie.....:
Raça.........:
Dt Nasciment: / /
Idade.......:
Sexo........: Não Informado

Pág.: 1 /1

MYCOPLASMA SP.

Material: Cultura de Células Coletado em: 12/07/2024 08:55 Método: PCR - Reação em Cadeia Polimerase

RESULTADO...........: NEGATIVO

NOTA................: Após as análises de DNA utilizando oligonucleotídeos específicos, foram determinados os resultados especificados.

OBSERVAÇÃO..........: 1.Os resultados obtidos se aplicam somente à amostra analisada.
2.Amostra coletada e enviada pelo solicitante.

VETERINÁRIO.........: "CONSULTE SEMPRE UM MÉDICO VETERINÁRIO DE SUA CONFIANÇA"

bio cell

Ensaio de Viabilidade Celular

Método: Azul de Trypan

BIOCELL 04.008.16 LT 190624.3

Meio: 08.24

Data: 30/07/2024

	30 min	
	Vivas	Mortas
Células	92,0%	8,0%

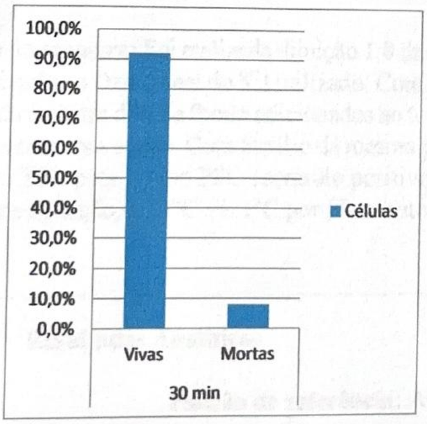

	24 Horas	
	Vivas	Mortas
Células	91,5%	8,5%

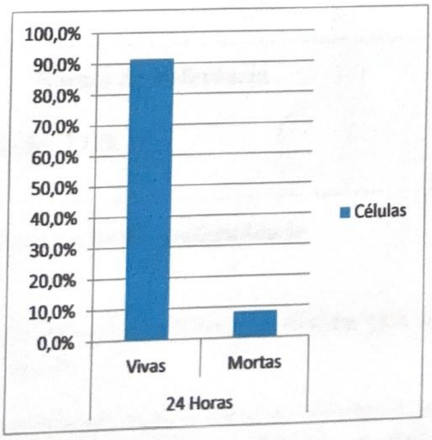

l Count Report Summary

rt date: 7/17/2024, 11:48:49 AM

Experiment Name	Biocell 04.008.16 LT 190624.3
Experiment Date	7/17/2024, 11:40:31 AM
Experiment By	Bio Cell, laboratorio@biocell.com.br
Gating	8 µm - 29 µm
Trypan Blue	0
Dilution	0
Algorithm Version	Cell Count - V3

Average cell size of (6) counts

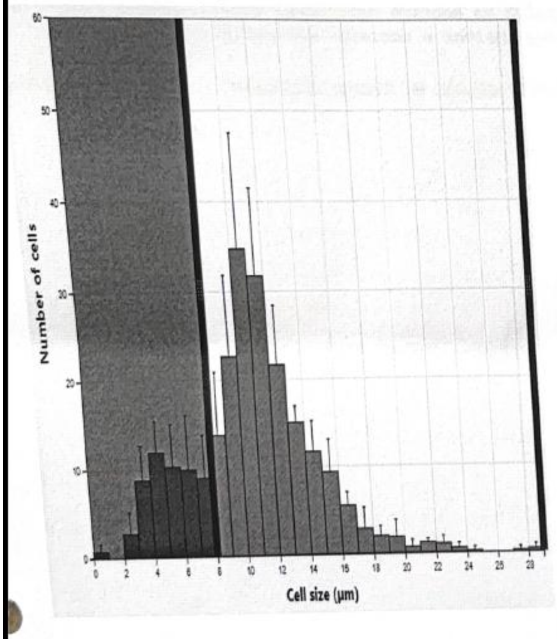

Average concentration of (6) counts

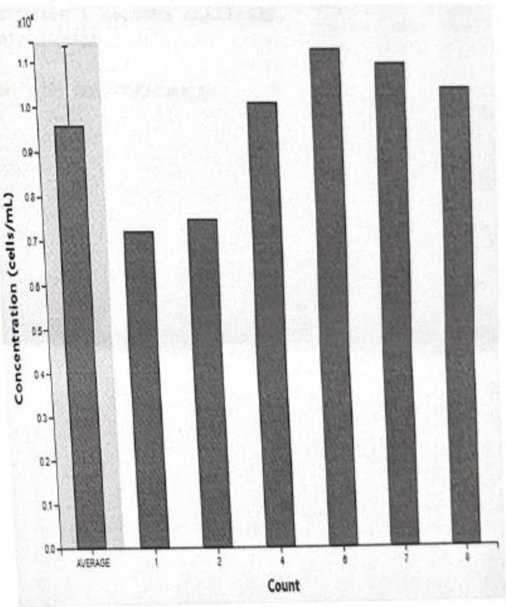

Average of 6 counts	
Gating	8 µm - 29 µm
Total cells	9.58e+5 ± 1.78e+5 cells/mL
Average Size	12.3 ± 0.6 µm

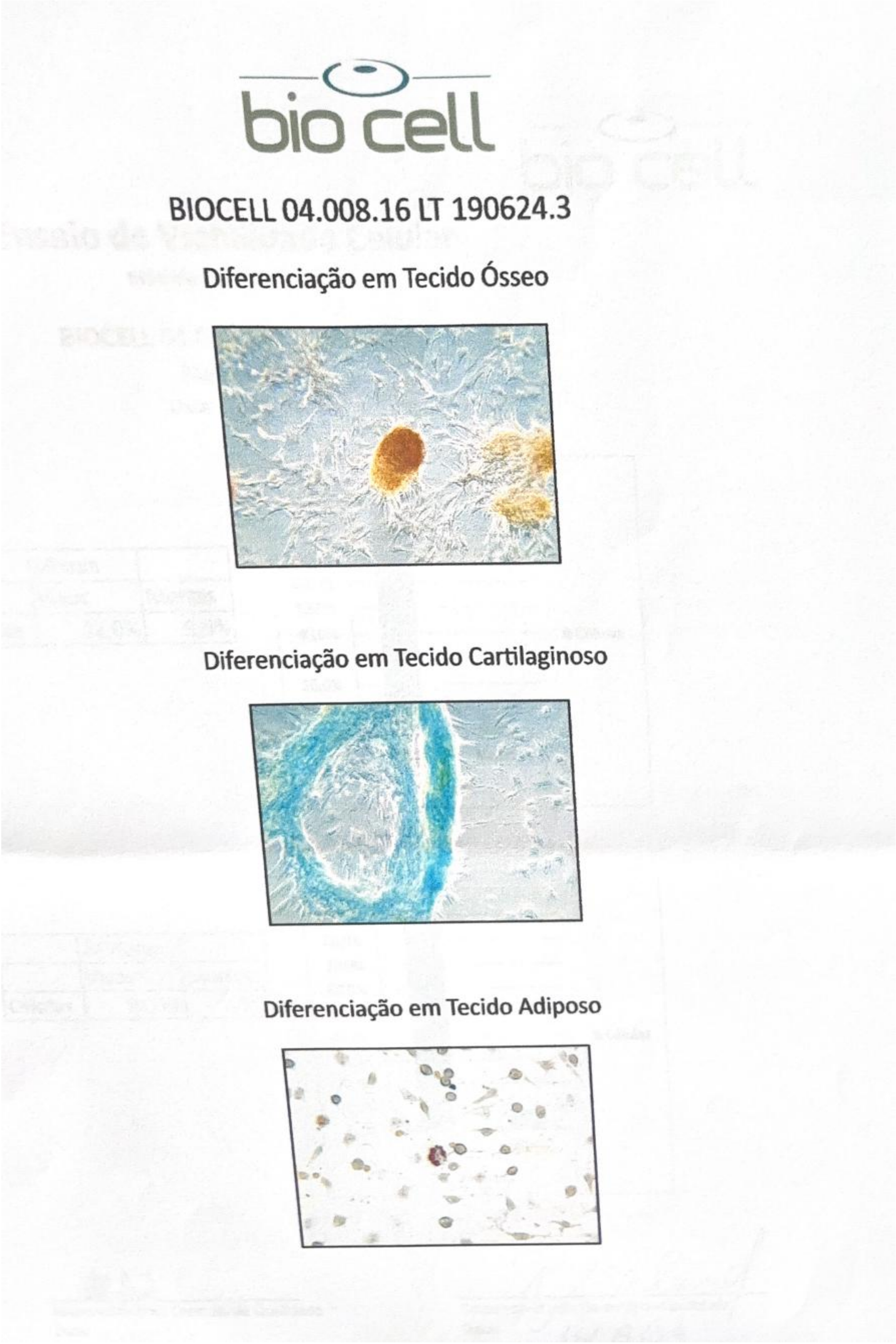

BIOCELL 04.008.16 LT 190624.3

Diferenciação em Tecido Ósseo

Diferenciação em Tecido Cartilaginoso

Diferenciação em Tecido Adiposo

Donor 2 – Female dog, 11 months, 4kg, Shih Tzu.

Batch identification: 04.002.21

HEALTH ASSESSMENT

SCINTE LABORATORIO VETERINÁRI

Paciente....:
Prop........:
Convênio....:
Méd.Vet.....:
Cadastro....:
Destino.....:

Anonymized

Requisição..: 698701
Espécie.....: Canina Filhote
Raça........: SHIH-TZU
Sexo........: Fêmea
Idade.......: 11 Mes(es)
Emissão.....: 14/06/2022 14:37

Pág.: 1 / 2

HEMOGRAMA COMPLETO

Material: Sangue total Coletado no cliente com entrada no laboratorio em: 20/04/2021 11:30 Método: Automatizado / pocH-100iV

Valores de Referência | Regua Refer

ERITROGRAMA

		Valores de Referência
Hemácias...................:	7,66 u³	04,60 a 06,00
Hemoglobina................:	17,90 g/dL	10,00 a 13,00
Hematócrito................:	47,10 %	30,00 a 39,00
Vol. Cor. Médio (VCM)......:	61,49 fL	65,00 a 73,00
H[illegible] Cor. Média (HCM)......:	23,37 pg	20,00 a 24,00
C[illegible]. Hemogl. (CHCM).......:	38,00 g/dL	30,00 a 36,00

Observação................:Hemacias Microcitica Normocromica
Presença de Acantócitos
Hemácias aglutinadas
Policitemia
Presença de Metarrubrícitos ++

LEUCOGRAMA

			Valores de Referência	
Leucócitos.................:	12.100 /mm³		9.000 a 16.000	
Pró-Mielócitos.............:	0 /mm³	0 %	0 a 0	0 a 0
Mieloblastos...............:	0 /mm³	0 %	0 a 0	0 a 0
Mielócitos.................:	0 /mm³	0 %	0 a 0	0 a 0
Metamielócitos.............:	0 /mm³	0 %	0 a 0	0 a 0
Bastonetes.................:	0 /mm³	0 %	0 a 320	0 a 2
Segmentados................:	6.413 /mm³	53 %	4.050 a 11.520	45 a 72
Eosinófilos................:	968 /mm³	8 %	180 a 1.600	2 a 10
Basófilos..................:	0 /mm³	0 %	0 a 320	0 a 2
Linfócitos.................:	4.598 /mm³	38 %	1.800 a 7.680	20 a 48
[illegible]ócitos Atípicos........:	0 /mm³	0 %	0 a 0	0 a 0
Monócitos..................:	121 /mm³	1 %	180 a 1.600	2 a 10
Linfoblastos...............:	0 /mm³	0 %	0 a 0	0 a 0
Monoblastos................:	0 /mm³	0 %	0 a 0	0 a 0
Blastos....................:	0 /mm³	0 %	0 a 0	0 a 0

Observação................:Leucócitos sem alterações Morfológicas

Plaquetas..................:	258.000 /mm³	150.000 a 500.000
Proteína Plasmática........:	7,00 g/dL	5,50 a 7,50

Observação................:Plasma Hemolisado

Os valores dos testes de laboratórios sofrem influência de estados fisiológicos, uso de medicamentos, incluindo regime alimentar e/ou tempo de jejum. Somente um profissional qualificado tem condições de interpretar corretamente estes resultados. Consulte sempre seu médico veterinário.

Anonymized

SANTÉ
LABORATÓRIO VETERINÁRI

Paciente....:
Prop........:
Convênio....: Anonymized
Méd.Vet.....:
Cadastro....:
Destino.....:

Requisição..: 698701
Espécie.....: Canina Filhote
Raça........: SHIH-TZU
Sexo........: Fêmea
Idade.......: 11 Mes(es)
Emissão.....: 14/06/2022 14:37

Pág.: 2/2

PROTEÍNAS TOTAIS E FRAÇÕES

Material: Soro Coletado no cliente com entrada no laboratorio em: 20/04/2021 11:30 Método: Ensaio colorimétrico

		Valores de Referência
Proteinas Totais...........:	6,62 g/dL	5,4 a 7,7
Albumina...................:	4,35 g/dL	2,3 a 3,8
Globulina..................:	2,27 g/dL	2,3 a 5,2
RELAÇÃO ALBUMINA/GLOBULINA.:	1,92 mg/dL	0,59 a 1,11
OBSERVAÇÃO.................:	Soro Hemolisado Exame confirmado.	

Regua Refer

EHRLICHIA CANIS+LYME+DIROFILARIA+ANAPLASMA

Material: SORO Coletado no cliente com entrada no laboratorio em: 20/04/2021 11:30 Método: ELISA

		Valores de Referência
EHRLICHIA CANIS......................:	NEGATIVO	Negativo
ANAPLASMA PHAGOCITOPHYLUM / A. PLATYS:	NEGATIVO	Negativo
DOENÇA DE LYME.......................:	NEGATIVO	Negativo
DIROFILARIA IMMITIS..................:	NEGATIVO	Negativo

Anonymized

bioinnova

LAUDO DE IMUNOFENOTIPAGEM

Método: fenotipagem por citometria de fluxo
Material: células-tronco mesenquimais
Lote: 04.002.21 LT170424.1

Marcadores	
CD90 (APC) - Invitrogen 17-5900-42	MARCADOR POSITIVO
CD44 (FITC) - BioLegend 103022	MARCADOR POSITIVO
CD29 (PerCP) - BioLegend 303024	MARCADOR POSITIVO
CD146 (FITC) - BioLegend 361012	MARCADOR NEGATIVO
MHCII (FITC) - BioRad MCA 1044P	MARCADOR NEGATIVO

RESULTADOS

CD90 Positivo	CD44 Positivo	CD29 Positivo	CD146 Negativo	MHCII Negativo
100%	100%	99,7%	0%	0,1%

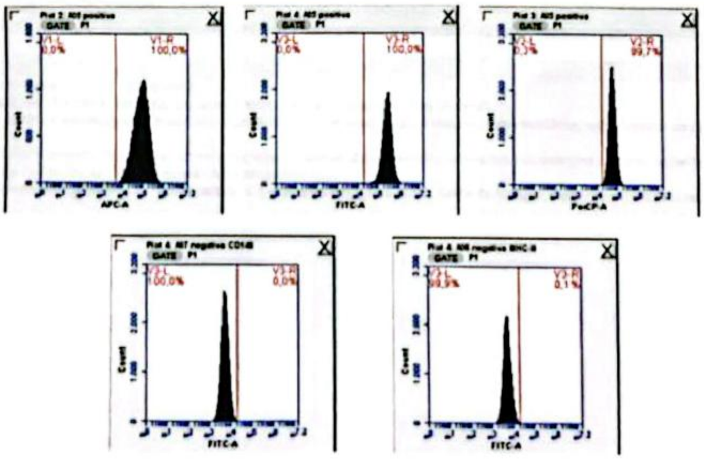

CONCLUSÃO

A população celular apresentou tripla positividade para os marcadores CD90, CD44 e CD29. Os resultados mostraram 100% da população marcada para CD90 e CD44 e 99,7% para CD29. Ainda, apresentou ausência de marcação de CD146 para 100% da população e para MHCII para 99,9%.

Hilana Brunel

Hilana dos Santos Sena Brunel
Gestora BioInnova

STERILITY TESTING

Relatório de Ensaio Nº 0000601933

[illegible]issão: **10/08/2024**

Identificação do Cliente

[illegible] BIO CELL TERAPIA CELULAR LTDA

[illegible]to:

[illegible]ereço: RUA 25 SUL BLOCO A LOJA 105, LOTE 30COMPLEMENTO :

[illegible]ADE ÁGUAS CLARAS

CNPJ/CPF: 19.283.681/0001-70

Telefone:(61) 999216776

Email:

Dados da Amostra: AMOSTRA 05: BIOCELL 04.002.21 LT 210624.1

Data de Coleta: 01/08/2024

Local da Coleta: -

Responsável Pela Coleta: Cliente

Data da Fabricação: 21/06/2024

Lote: 210624.1

Anonymized

Informações gerais: COLETADO POR:

Plano de amostragem: Plano de amostr[illegible]

Data Recebimento: 02/08/2024 12:50:00

Hora Coleta: 11:00:00

Quantidade: 10 mL

Data de Validade: -

Temperatura coleta(°C): AMBIENTE

Temperatura recebimento(°C): 10.6

Resultados Analíticos

Ensaio(s)	Resultado(s)	Unidade	Valor de referência	Metodologia(s)	Data Ensaio(s)
Teste de esterilidade - IN SITU	SEM CRESCIMENTO	-	Sem crescimento	FARMACOPEIA BRASILEIRA – VOLUME 1 – MÉTODO – 5.5.3.2.1. 2019	

Norma de referência

Declaração de Conformidade

A presente amostra atende aos padrões estabelecidos pela legislação vigente conforme norma de referência citada.

Os resultados obtidos foram comparados com o valor de referência de acordo com a legislação solicitada. Os resultados com valores acima do valor de referência foram considerados resultados não conforme.

Os resultados quantitativos, quando expressos, não consideram a incerteza inerente aos métodos analíticos como regra para decisão quanto à conformidade a uma especificação ou norma.

O(s) ensaio(s) sem valor(es) de referência estabelecido devem ser avaliados tecnicamente pelo responsável da empresa.

Opiniões e Interpretações

"As opiniões e interpretações expressas abaixo não fazem parte do escopo da acreditação deste laboratório"

NOTAS

- Estes resultados se referem apenas à amostra analisada.
- Os serviços são realizados nas instalações permanentes do laboratório, exceto os descritos como "In Loco".
- Na coleta realizada pelo cliente, a amostragem é de sua responsabilidade e as amostras serão analisadas como recebidas, salvo quando as mesmas não apresentarem condições.
- É responsabilidade do cliente o preenchimento adequado na solicitação de análise, das informações referentes aos dados da amostra, incluindo data e hora de coleta, data de fabricação e validade, lote e responsável e local da amostragem.
- Este relatório somente poderá ser reproduzido em sua totalidade. O Laboratório F.A.Z Análises se isenta de qualquer responsabilidade pela reprodução parcial do mesmo.

Teste de Endotoxinas

Amostra: BIO CELL 04.002.21 LT 210624.1
Data de Realização do Teste: 24/07/2024
Kit de Testagem: Teste LAL – *Limulus* Amebocyte Lysate PYROSATE®
Código: PSD030-30 **Sensibilidade:** 0,03 EU/mL (λ)
Lote: PS22001-30 **Validade:** 04 NOV 2024

Descrição do Processamento da amostra: Foi realizada diluição 1:8 da amostra em água apirogênica, conforme preconizado no Data Sheet do Kit utilizado. Com auxílio de pipeta de bulbo apirogênica, 500 µL da amostra diluída foram adicionados ao tubo SPL (amostra teste) e esse foi agitado gentilmente com a mão. Com auxílio da mesma pipeta transferiu-se 250µL do conteúdo do tubo SPL para o tubo PPC (controle positivo). Ambos foram incubados em banho maria, sem agitação, a 37°C +/- 1°C por 58 minutos para análise de resultado.

Resultados Analíticos

Resultado: Amostra Líquida **Padrão de referência:** Amostra Líquida

Metotolodgia: Farmacopéia Brasileira – Método 5.5.2.2 Endotoxinas bacterianas. 2019

Norma de Referência

Farmacopeia Brasileira, 6° Edição. 2019.

Declaração de conformidade

A amostra analisada está dentro dos padrões estabelecidos pela legislação vigente conforme norma de referência citada.

O resultado final foi comparado com o valor de referência de acordo com a legislação solicitada. Tratando-se de uma análise qualitativa, resultados diferentes do padrão de referência serão considerados resultados não conformes.

bio cell

Ensaio de Viabilidade Celular

Método: Azul de Trypan

BIOCELL 04.002.21 LT 210624.1

Meio: 03.24

Data: 06/08/2024

	30 min	
	Vivas	Mortas
Células	94,5%	5,5%

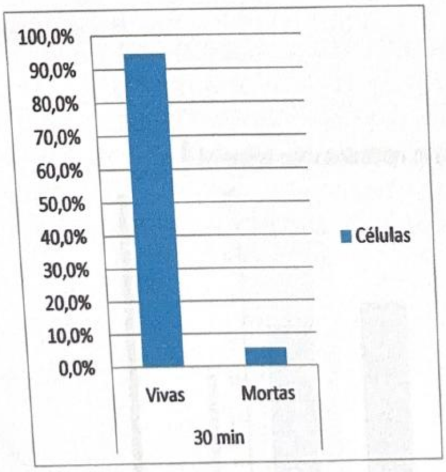

	24 Horas	
	Vivas	Mortas
Células	83,5%	16,5%

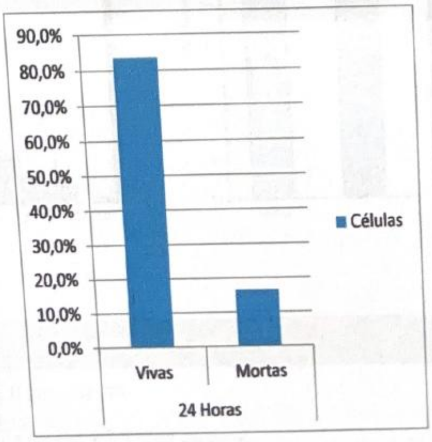

Count Report Summary

date: 7/30/2024, 3:10:07 PM

Experiment Name	BIOCELL 04.002.21 LT 210624.1
Experiment Date	7/30/2024, 3:04:44 PM
Experiment By	Bio Cell, laboratorio@biocell.com.br
Gating	8 µm - 33 µm
Trypan Blue	0
Dilution	0
Algorithm Version	Cell Count - V3

erage cell size of (4) counts

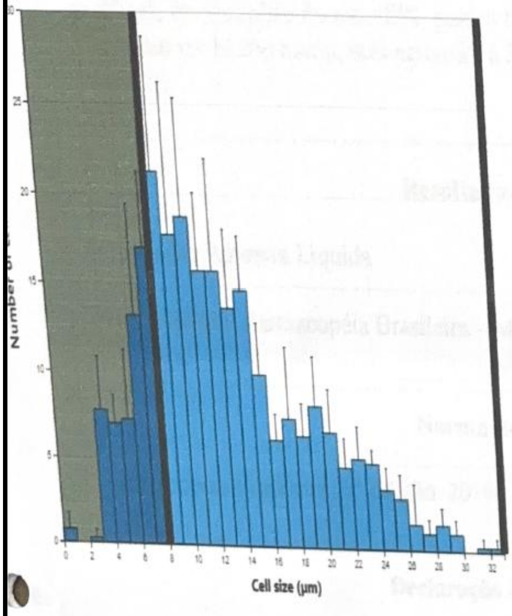

Average concentration of (4) counts

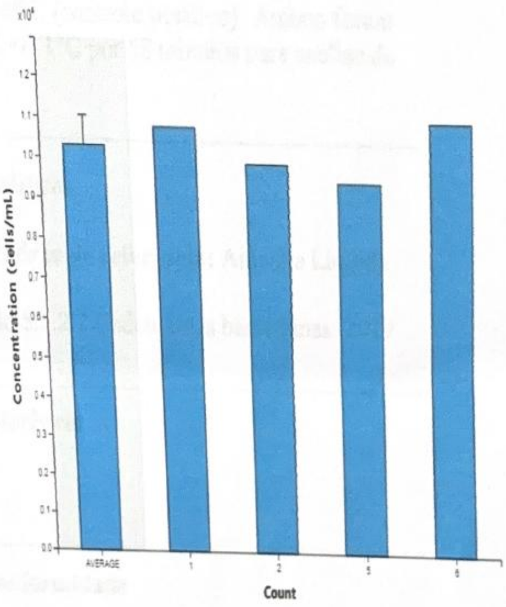

Average of 4 counts	
Gating	8 µm - 33 µm
Total cells	1.03e+6 ± 7.17e+4 cells/mL
Average Size	14.7 ± 1.1 µm

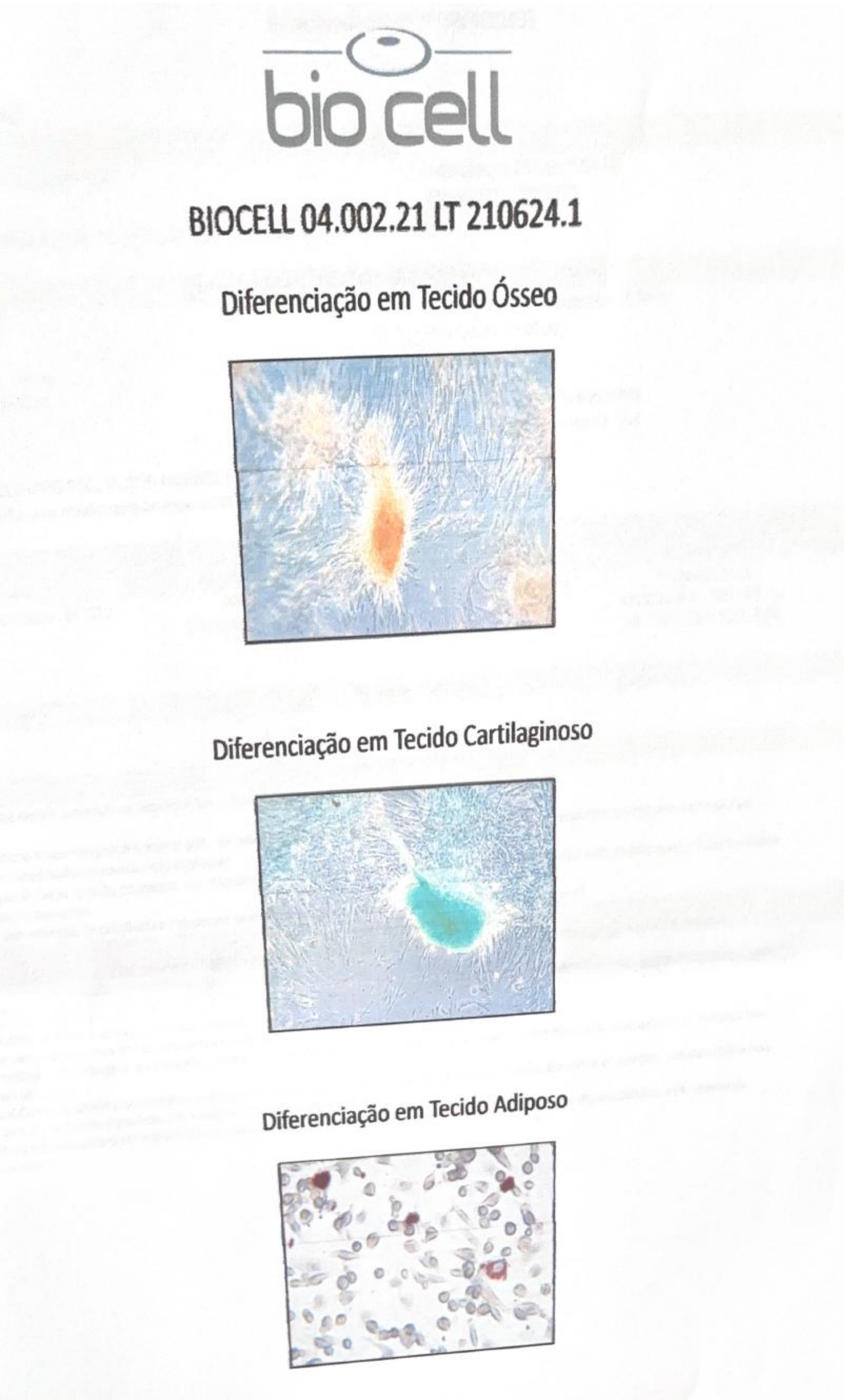

bio cell

BIOCELL 04.002.21 LT 210624.1

Diferenciação em Tecido Ósseo

Diferenciação em Tecido Cartilaginoso

Diferenciação em Tecido Adiposo

108

Donor 3 – Female dog, 4 years, 30kg, Mixed breed.

Batch identification: 04.004.23

HEALTH ASSESSMENT

SANTÉ
LABORATÓRIO VETERINÁRIO
20 ANOS

aciente....:
rop........:
onvênio....:
éd.Vet.....:
adastro....:
estino.....:

Anonymized

Requisição..: 877049
Espécie.....: Canina
Raça........: SRD
Sexo........: Fêmea
Idade.......:
Emissão.....: 20/09/2023 08:21

Pág.: 1 / 4

HEMOGRAMA COMPLETO

aterial: Sangue total Coletado no cliente com entrada no laboratorio em: 06/09/2023 17:58 Método: Automatizado / pocH-100iV

Valores de Referência — Regua Referenci

ERITROGRAMA

		Valores de Referência
Hemácias	7,15 u³	05,50 a 08,50
Hemoglobina	17,10 g/dL	12,00 a 18,00
Hematócrito	54,20 %	37,00 a 55,00
Vol. Cor. Médio (VCM)	75,80 fL	60,00 a 77,00
Hem. Cor. Média (HCM)	23,92 pg	19,00 a 23,00
C[illegible] Hemogl. (CHCM)	31,55 g/dL	31,00 a 37,00
RDW - CV	13,60 %	12,00 a 15,00

Observação.................:Hemacias Normocitica Normocromica

LEUCOGRAMA

			Valores de Referência	
Leucócitos	5.300 /mm³		6.000 a 17.000	
Monoblastos	0 /mm³	0 %	0 a 0	0 a 0
Pró-Mielócitos	0 /mm³	0 %	0 a 0	0 a 0
Mieloblastos	0 /mm³	0 %	0 a 0	0 a 0
Mielócitos	0 /mm³	0 %	0 a 0	0 a 0
Metamielócitos	0 /mm³	0 %	0 a 170	0 a 1
Bastonetes	0 /mm³	0 %	0 a 510	0 a 3
Segmentados	3.657 /mm³	69 %	3.600 a 13.090	60 a 77
Eosinófilos	265 /mm³	5 %	0 a 1.700	0 a 10
Basófilos	0 /mm³	0 %	0 a 340	0 a 2
Linfócitos	1.325 /mm³	25 %	720 a 5.100	12 a 30
Linfócitos Reativo	0 /mm³	0 %	0 a 170	0 a 1
Monócitos	53 /mm³	1 %	0 a 1.700	0 a 10
Li[illegible]foblastos	0 /mm³	0 %	0 a 0	0 a 0
B[illegible]tos	0 /mm³	0 %	0 a 0	0 a 0

Observação.................:Leucopenia
Leucócitos sem alterações Morfológicas

		Valores de Referência
Plaquetas	180.000 /mm³	175.000 a 500.000
MPV	9,70 fL	7,44 a 10,71
PDW	15,60 %	15,32 a 16,82
Proteina Plasmática	6,60 g/dL	6,00 a 8,00

Observação.................:Plasma Hemolisado
Plasma Lipêmico

Os resultados dos testes laboratoriais sofrem influências de estados fisiológicos, patológicos, uso de medicamentos, etc. A interpretação desta análise e a conclusão diagnosticada é um ato médico veterinário e depende da análise conjunta dos dados clínicos e epidemiológico.
O Santé Laboratório possui assessoria científica qualificada para discussão de resultados com Médico Vet. solicitante.
O presente resultado refere-se exclusivamente à amostra enviada ao Santé Laboratório.

Anonymized

SANTÉ
LABORATÓRIO VETERINÁRIO

Paciente....:
Prop........:
Convênio....: Anonymized
Méd.Vet.....:
Cadastro....:
Destino.....:

Requisição..: 877049
Espécie.....: Canina
Raça........: SRD
Sexo........: Fêmea
Idade.......:
Emissão.....: 20/09/2023 08:21

EHRLICHIA CANIS+LYME+DIROFILARIA+ANAPLASMA

Material: SORO Coletado no cliente com entrada no laboratorio em: 06/09/2023 17:58 Método: ELISA

		Valores de Referência
EHRLICHIA CANIS......................:	Negativo	Negativo
ANAPLASMA PHAGOCITOPHYLUM / A. PLATYS:	Negativo	Negativo
DOENÇA DE LYME.......................:	Negativo	Negativo
DIROFILARIA IMMITIS..................:	Negativo	Negativo

NOTA.................................: Os resultados dos testes laboratoriais sofrem influências de estados fisiológicos, patológicos, uso de medicamentos, etc. A interpretação desta análise e a conclusão diagnosticada é um ato médico veterinário e depende da análise conjunta dos dados clínicos e epidemiológico.
O Santé Laboratório possui assessoria científica qualificada para discussão de resultados com Médico Vet. solicitante.
O presente resultado refere-se exclusivamente à amostra enviada ao Santé Laboratório.

Anonymized

113

SANTÉ
LABORATÓRIO VETERINÁRIO

Paciente....:	Anonymized	Requisição..:	877049
Prop........:		Espécie.....:	Canina
Convênio....:		Raça........:	SRD
Méd.Vet.....:		Sexo........:	Fêmea
Cadastro....:		Idade.......:	
Destino.....:		Emissão.....:	20/09/2023 08:21

Pág.: 4 / 4

LEISHMANIOSE (ESQUEMA VACINAL)

Material: SORO Coletado no cliente com entrada no laboratorio em: 06/09/2023 18:46 Método: Imunocromatográfico

Valores de Referência

RESULTADO...........: Negativo

LOTE................: 202301001V
VALIDADE............: 30/04/2025
NOTA................: - O exame está sujeito, embora raramente, a ocorrência de resultados falso- negativo e falso-positivo, que é uma caracteristica de variações pre- analiticas e das metodologias. Sugerimos o acompanhamento médico veterinário da sintomatologia clínica.
- Um cão para ser considerado um caso confirmado para leishmaniose visceral deverá apresentar resultados reagentes nos seguintes ensaios sorológicos: teste rápido imunocromatográfico (TRI) e ensaio imunoenzimático (ELISA).
- Os resultados dos testes sorológicos não reagentes são considerados válidos por, no máximo, 30 dias contados da data da coleta da amostra.

KIT COM LICENÇA NO MINISTÉRIO DA AGRICULTURA - MAPA N° 10.357/2020

Exame realizado no Santé Laboratorio.

Anonymized

bioinnova

Email: laboratorio@biocell.com.br
End: R. 25 Sul, lote 30 - Águas Claras/DF
Telefone: (61) 9 9921-6776

Data: 29/09/2023
Responsável Técnico: Karine R D Silveira

Identificação BIOCELL 04.004.23 LT 180923.2

Laudo de Imunofenotipagem

Método: Imunofenotipagem por citometria de fluxo
Material: Células-tronco mesenquimais

Marcador

CD90 (PE)	MARCADOR POSITIVO	BD - LOTE 202304
CD44 (FITC)	MARCADOR POSITIVO	BioLegend - LOTE B277911
CD29 (PerCP)	MARCADOR POSITIVO	BioLegend- LOTE B346050
MHCII (FITC)	MARCADOR NEGATIVO	BioRad - LOTE 155329

Resultados

Triple Positive	Triple Negative	Single Positive	Single Positive	Single Positive	Single Positive	Single Negative
CD44/ CD29/ CD90	CD44/ CD29/ CD90	CD44	CD29	CD90	MHC II	MHC II
80,09 %	0,00%	19,01%	0,00%	0,01%	0,04%	99,92%

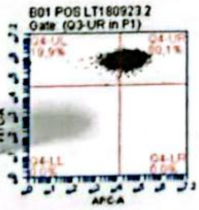

Plot FITC X PerCP-A: Marcadores CD44/CD29/CD90.

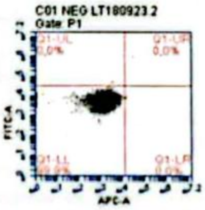

Plot FITC X PerCP-A: Marcador MHCII.

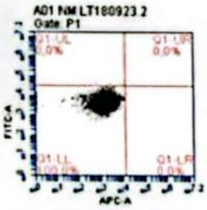

Plot FITC X PerCP-A: Sem Marcador.

Descritivo

Expressão dos marcadores positivos de células-tronco CD29, CD44 e CD90 na superfície celular em 80,09% do plot amostral analisado. Expressão do marcador negativo de células-tronco MHCII em 0,04% do plot amostral analisado.

Conclusão

Em função somatória dos fatos analisados, o grupo amostral refere-se às células-tronco mesenquimais. Células se encontram aptas para uso.

Karine Silveira	Kailana Brunel	[illegible]
Responsável Técnico	Responsável pela Garantia da Qualidade	Responsável pelo Controle da Qualidade
Data: 03/10/23	Data: 03/10/2023	Data: 03/10/23

Relatório de Ensaio Nº 0000601929

são: 10/08/2024

**Identificação do Cliente**	
CELL TERAPIA CELULAR LTDA	**CNPJ/CPF:** 19.283.681/0001-70<br>**Telefone:**(61) 999216776
UA 25 SUL BLOCO A LOJA 105, LOTE 30COMPLEMENTO :<br>JAS CLARAS	**Email:**

**Dados da Amostra: AMOSTRA 01: BIOCELL 04.004.23 LT 120624.1**	
**eta:** 01/08/2024<br>**leta:** -<br>**el Pela Coleta:** Cliente<br>**bricação:** 12/06/2024<br>24.1	**Data Recebimento:** 02/08/2024 12:50:00<br>**Hora Coleta:** 11:00:00<br>**Quantidade:** 10 mL<br>**Data de Validade:** -<br>**Temperatura coleta(°C):** AMBIENTE<br>**Temperatura recebimento(°C):** 10.6

es gerais: COLETADO POR: \ Anonymized
amostragem: Plano de amostra

Resultados Analíticos

Ensaio(s)	Resultado(s)	Unidade	Valor de referência	Metodologia(s)	Data Ensaio(s)
Teste de esterilidade - IN SITU	SEM CRESCIMENTO	-	Sem crescimento	FARMACOPEIA BRASILEIRA – VOLUME 1 – MÉTODO – 5.5.3.2.1. 2019	02/08/2024

Norma de referência

COPÉIA BRASILEIRA, 6ª EDIÇÃO, 2019

Declaração de Conformidade

ente amostra atende aos padrões estabelecidos pela legislação vigente conforme norma de referência citada.

ultados obtidos foram comparados com o valor de referência de acordo com a legislação solicitada. Os resultados com valores acima do valor de
ncia foram considerados resultados não conforme.
sultados quantitativos, quando expressos, não consideram a incerteza inerente aos métodos analíticos como regra para decisão quanto à conformidade
especificação ou norma.
ensaio(s) sem valor(es) de referência estabelecido devem ser avaliados tecnicamente pelo responsável da empresa.

Opiniões e Interpretações

"As opiniões e interpretações expressas abaixo não fazem parte do escopo da acreditação deste laboratório"

NOTAS

tes resultados se referem apenas à amostra analisada.
serviços são realizados nas instalações permanentes do laboratório, exceto os descritos como "In Loco".
coleta realizada pelo cliente, a amostragem é de sua responsabilidade e as amostras serão analisadas como recebidas, salvo quando as mesmas não
esentarem condições.
responsabilidade do cliente o preenchimento adequado na solicitação de análise, das informações referentes aos dados da amostra, incluindo data e hora
coleta, data de fabricação e validade, lote e responsável e local da amostragem.
ste relatório somente poderá ser reproduzido em sua totalidade. O Laboratório F.A.Z Análises se isenta de qualquer responsabilidade pela reprodução
rcial do mesmo.

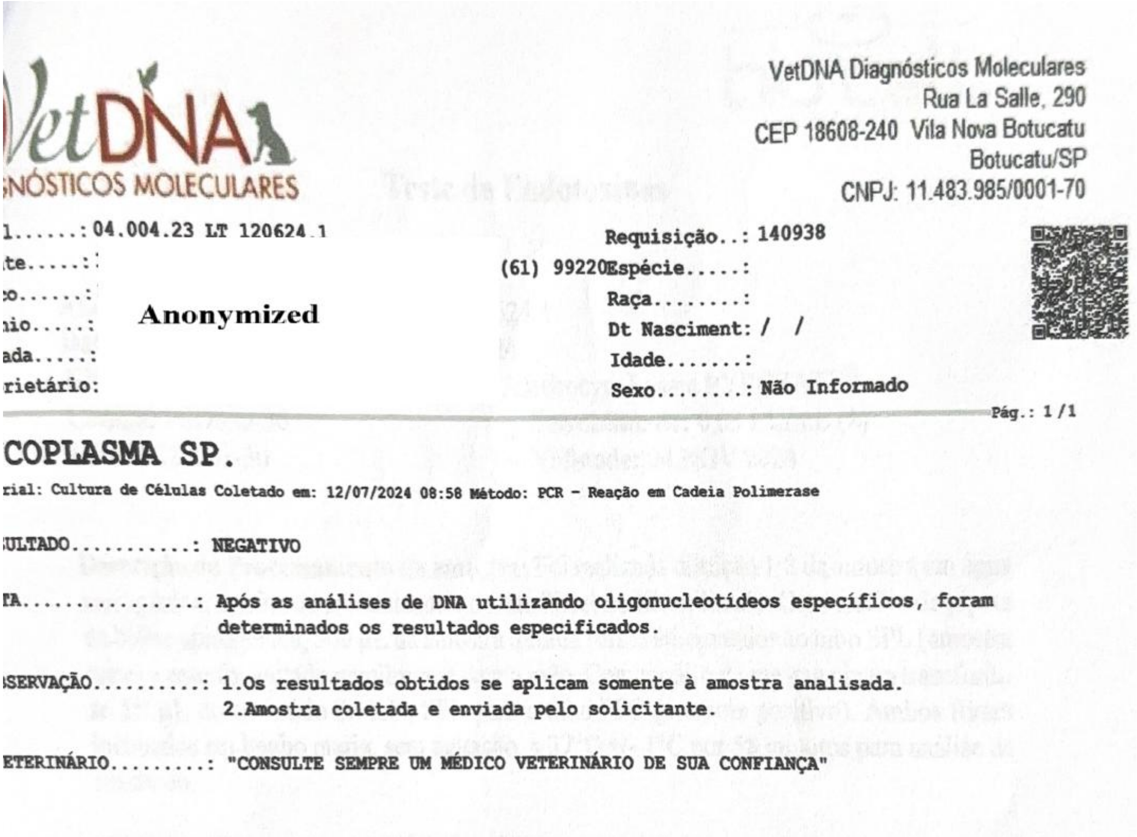
VetDNA
DIAGNÓSTICOS MOLECULARES

VetDNA Diagnósticos Moleculares
Rua La Salle, 290
CEP 18608-240 Vila Nova Botucatu
Botucatu/SP
CNPJ: 11.483.985/0001-70

l......: 04.004.23 LT 120624.1
ite.....:
io......:
nio.....: Anonymized
ada.....:
rietário:

(61) 99220

Requisição..: 140938
Espécie.....:
Raça........:
Dt Nasciment: / /
Idade.......:
Sexo........: Não Informado

Pág.: 1 /1

COPLASMA SP.

rial: Cultura de Células Coletado em: 12/07/2024 08:58 Método: PCR - Reação em Cadeia Polimerase

ULTADO...........: NEGATIVO

TA................: Após as análises de DNA utilizando oligonucleotídeos específicos, foram determinados os resultados especificados.

SERVAÇÃO..........: 1.Os resultados obtidos se aplicam somente à amostra analisada.
2.Amostra coletada e enviada pelo solicitante.

ETERINÁRIO.........: "CONSULTE SEMPRE UM MÉDICO VETERINÁRIO DE SUA CONFIANÇA"

bio cell

Teste de Endotoxinas

Amostra: BIO CELL 04.004.23 LT 120624.1
Data de Realização do Teste: 10/07/2024
Kit de Testagem: Teste LAL – *Limulus* Amebocyte Lysate PYROSATE®
Código: PSD030-30
Lote: PS22001-30
Sensibilidade: 0,03 EU/mL (λ)
Validade: 04 NOV 2024

Descrição do Processamento da amostra: Foi realizada diluição 1:8 da amostra em água apirogênica, conforme preconizado no Data Sheet do Kit utilizado. Com auxílio de pipeta de bulbo apirogênica, 500 μL da amostra diluída foram adicionados ao tubo SPL (amostra teste) e esse foi agitado gentilmente com a mão. Com auxílio da mesma pipeta transferiu-se 250μL do conteúdo do tubo SPL para o tubo PPC (controle positivo). Ambos foram incubados em banho maria, sem agitação, a 37°C +/- 1°C por 58 minutos para análise de resultado.

Resultados Analíticos

Resultado: Amostra Líquida **Padrão de referência:** Amostra Líquida

Metotolodgia: Farmacopéia Brasileira – Método 5.5.2.2 Endotoxinas bacterianas. 2019

Norma de Referência

Farmacopeia Brasileira, 6° Edição. 2019.

Declaração de conformidade

A amostra analisada está dentro dos padrões estabelecidos pela legislação vigente conforme norma de referência citada.

O resultado final foi comparado com o valor de referência de acordo com a legislação solicitada. Tratando-se de uma análise qualitativa, resultados diferentes do padrão de referência serão considerados resultados não conformes.

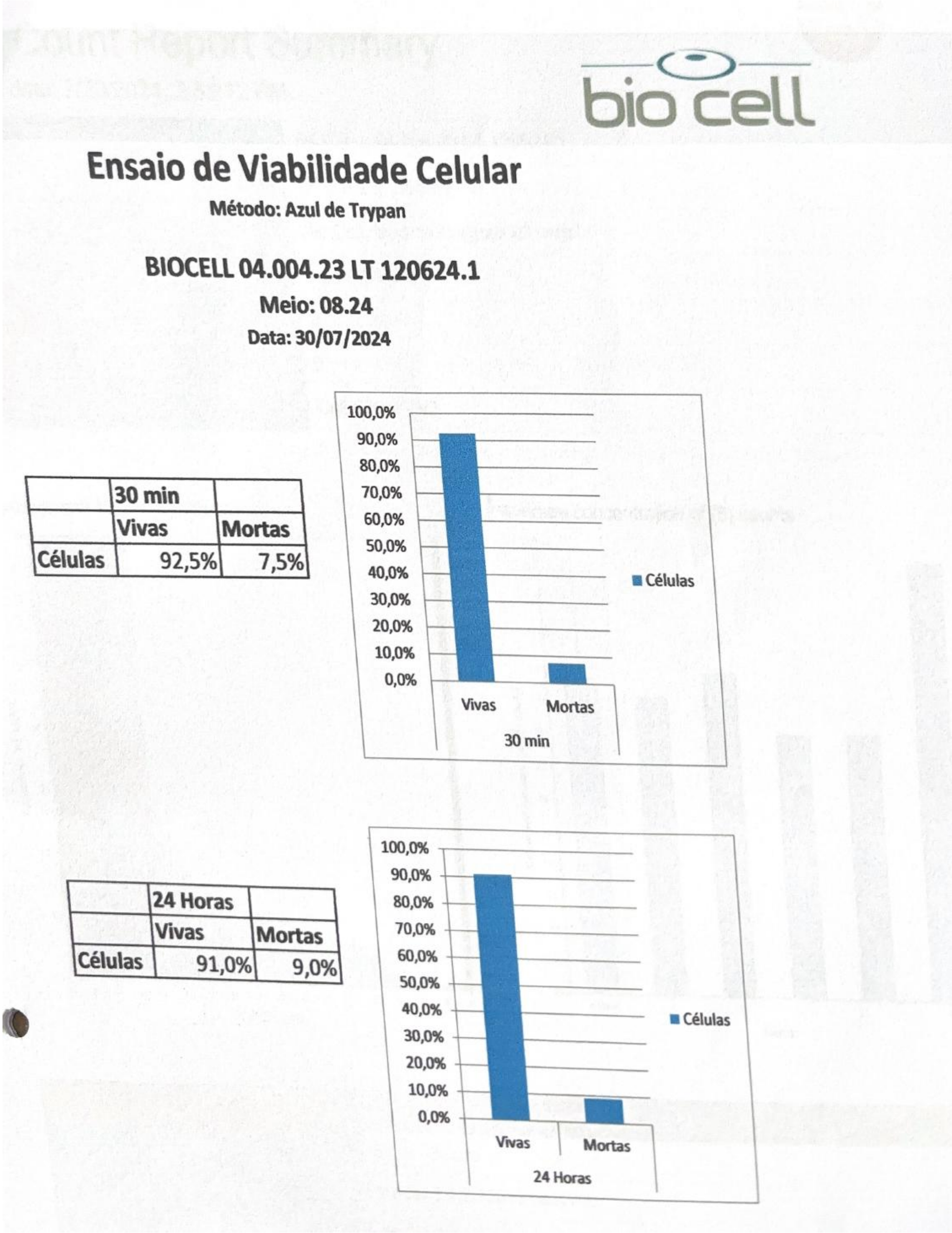

bio cell

Ensaio de Viabilidade Celular

Método: Azul de Trypan

BIOCELL 04.004.23 LT 120624.1

Meio: 08.24

Data: 30/07/2024

	30 min	
	Vivas	Mortas
Células	92,5%	7,5%

	24 Horas	
	Vivas	Mortas
Células	91,0%	9,0%

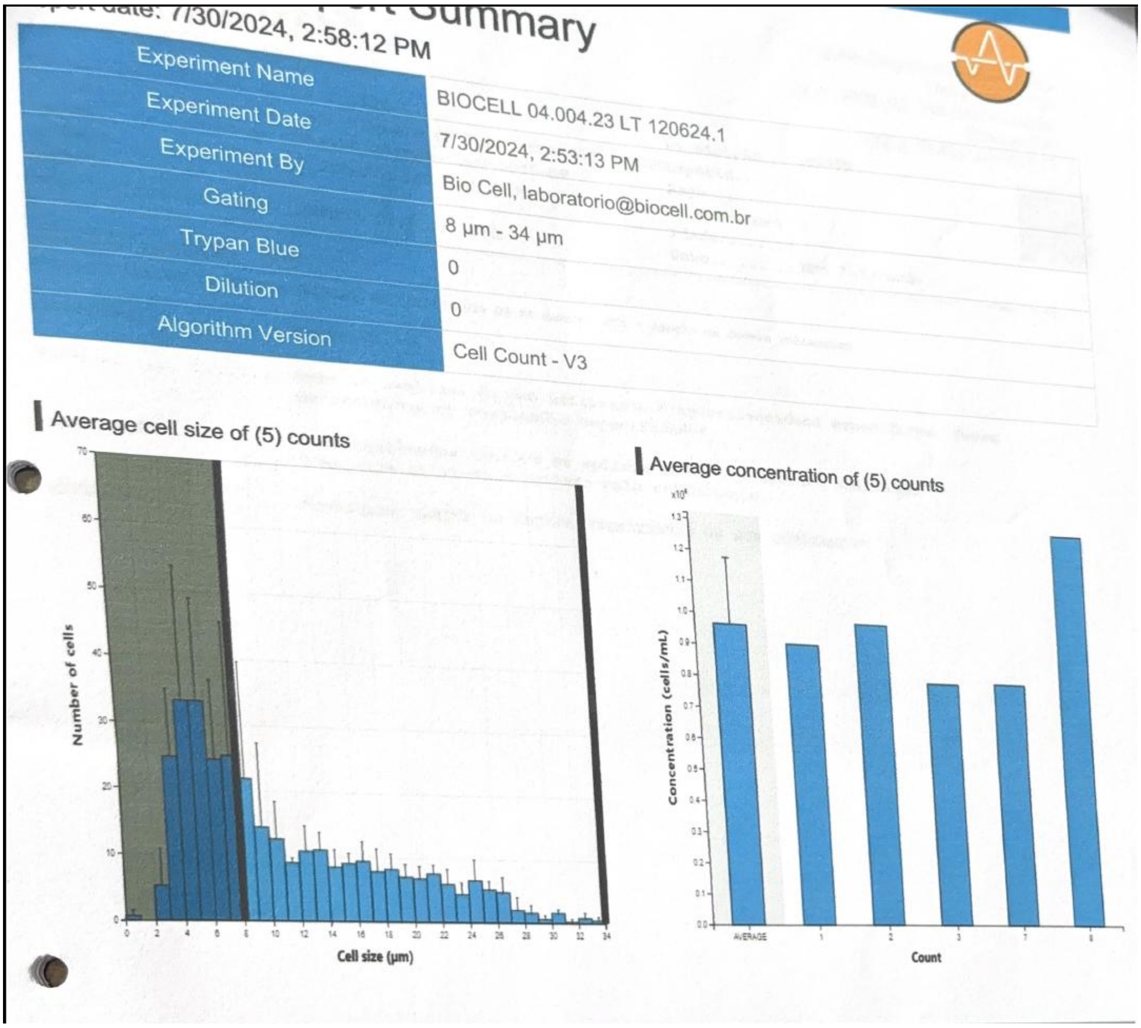

7/30/2024, 2:58:12 PM
Summary
Experiment Name
BIOCELL 04.004.23 LT 120624.1
Experiment Date
7/30/2024, 2:53:13 PM
Experiment By
Bio Cell, laboratorio@biocell.com.br
Gating
8 µm - 34 µm
Trypan Blue
0
Dilution
0
Algorithm Version
Cell Count - V3
Average cell size of (5) counts
Number of cells
Cell size (µm)
Average concentration of (5) counts
Concentration (cells/mL)
AVERAGE
Count

bio cell

BIOCELL 04.004.23 LT 120624.1

Diferenciação em Tecido Ósseo

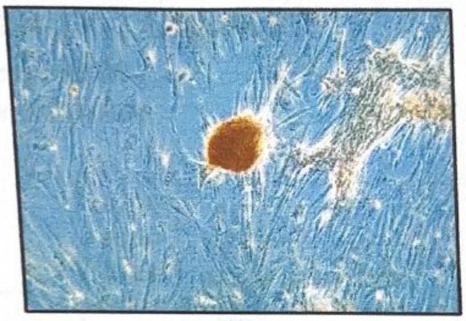

Diferenciação em Tecido Cartilaginoso

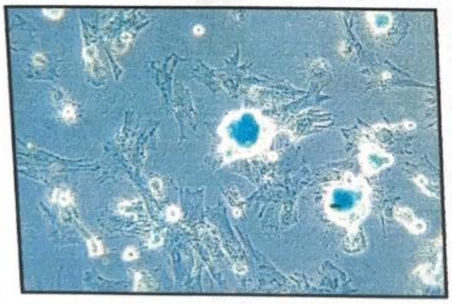

Diferenciação em Tecido Adiposo

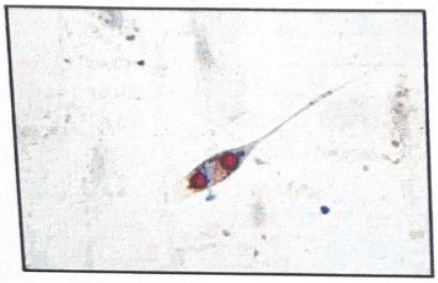

Supplement: Supplementary file 1 — Supplementary Material 1 (PDF 3.86 MB) [file 11259_2026_11343_MOESM1_ESM.pdf]
